# Enhancing Lithium-Sulfur Battery Performance by MXene, Graphene, and Ionic Liquids: A DFT Investigation

**DOI:** 10.3390/molecules29010002

**Published:** 2023-12-19

**Authors:** Jianghui Cao, Sensen Xue, Jian Zhang, Xuefeng Ren, Liguo Gao, Tingli Ma, Anmin Liu

**Affiliations:** 1 State Key Laboratory of Fine Chemicals, School of Chemical Engineering, Dalian University of Technology, Panjin 124221, China; cjh18235166001@outlook.com (J.C.); 18342782557@163.com (J.Z.);; 2 School of Ocean Science and Technology, Dalian University of Technology, Panjin 124221, China; 3 Department of Materials Science and Engineering, China Jiliang University, Hangzhou 310018, China; tinglima@life.kyutech.ac.jp; 4 Graduate School of Life Science and Systems Engineering, Kyushu Institute of Technology, 2-4 Hibikino, Wakamatsu, Fukuoka 808-0196, Japan

**Keywords:** d-Ti_3_C_2_ MXene, ionic liquids, polysulfides dissolution, graphene oxide, density functional theory

## Abstract

The efficacy of lithium-sulfur (Li-S) batteries crucially hinges on the sulfur immobilization process, representing a pivotal avenue for bolstering their operational efficiency and durability. This dissertation primarily tackles the formidable challenge posed by the high solubility of polysulfides in electrolyte solutions. Quantum chemical computations were leveraged to scrutinize the interactions of MXene materials, graphene (Gr) oxide, and ionic liquids with polysulfides, yielding pivotal binding energy metrics. Comparative assessments were conducted with the objective of pinpointing MXene materials, with a specific focus on d-Ti_3_C_2_ materials, evincing augmented binding energies with polysulfides and ionic liquids demonstrating diminished binding energies. Moreover, a diverse array of Gr oxide materials was evaluated for their adsorption capabilities. Scrutiny of the computational outcomes unveiled an augmentation in the solubility of selectively screened d-Ti_3_C_2_ MXene and ionic liquids—vis à vis one or more of the five polysulfides. Therefore, the analysis encompasses an in-depth comparative assessment of the stability of polysulfide adsorption by d-Ti_3_C_2_ MXene materials, Gr oxide materials, and ionic liquids across diverse ranges.

## 1. Introduction

Even though lithium-ion batteries (LIBs) have been a major success in portable devices and electric vehicles, more energy density is still necessary. The intrinsically determined topological intercalation chemistry of LIBs during the past three decades has almost reached its predicted energy density limit [1,2,3,4,5,6,7]. Based on its cathodic conversion reaction and anodic deposition/depletion mechanism, Li-S batteries are potential replacement candidates since their energy density is around six times larger than LIBs. Regarding the application prospect of Li-S batteries, the high theoretical specific capacity is 1675 Ah/kg, and the high energy density of sulfur is 2600 Wh/kg [8,9]. The advantage of Li-S batteries is further supported by the non-toxicity, affordability, and environmental friendliness of sulfur as a cathode material [10,11,12,13,14,15]. Furthermore, Li-S cells offer a notable benefit over Li-ion cells in terms of user-level maintenance in that they do not need top-up charging when in storage.

However, the design of high sulfur cathodes, the suppression of the shuttle effect, the growth of dendrites, the improvement of performance, and large-scale preparation still face many challenges [16,17,18,19,20,21,22,23]. Moreover, the poor conductivity of sulfur, as well as its easy dissolution in the electrolyte solution, is one of the main problems faced, which leads to poor charging kinetics and low sulfur utilization in the battery. Thirdly, the shuttle movement of long-chain polysulfides, denoted as Li_2_S_n_ (4 ≤ *n* ≤ 8), represents a soluble intermediate product. This phenomenon leads to notable capacity degradation and a shortened cycle life [10,11,24]. Additionally, the discharge medium S_x_^2−^ is easy to dissolve in the organic electrolyte, which has a shuttling effect, leading to lower coulombic efficiency and faster capacity decay, and the volume change during the discharge effect has the potential to destroy the electrode structure, and the volume change of the sulfur cathode is almost more than 80% during the charge/discharge cycle, which greatly reduces the cycling performance [16,18,25]. In recent years, the discharge mechanism of lithium batteries has been investigated using density functional theory (DFT) and molecular dynamics. In the discharge process, sulfur, which first takes the form of a ring-shaped rhombic S_8_ molecule by ring cleaving, is reduced to S_8_^2−^ while lithium metal is immediately oxidized into Li^+^. The subsequent reduction is S_8_^2−^ to S_4_^2−^ and eventually S_2_^2−^/S^2−^. On the cathode side, S^2−^ undergoes reversible oxidation to become S_2_^2−^, S_4_^2−^, S_8_^2−^, and S_8_ during charging, while Li metal is deposited on the anode side. Therefore, the retention of S_8_ is important for the long-term performance of lithium batteries, however, it is not clear whether polar groups can promote the adsorption of nonpolar S_8_ when interacting with lithium polysulphide, and whether functional groups can form a barrier to electron transfer at the interface between S_8_ and the substrate [26,27,28].

To alleviate the dissolution effect in the battery from the point of cathode material modification, Tao et al. proved that MgO, CeO_2_, and La_2_O_3_ show higher capacity with the use of DFT calculations, which revealed that better surface diffusion leads to higher deposition efficiency of sulfide species [16]. Kim et al. indicated that Li_2_S_2_ exhibits better redox activities compared to Li_2_S, which influences the reversibility, with the combination of DFT calculations and experiments. These results indicate that Li_2_S_2_ exhibits better redox activities compared to Li_2_S [12]. The modified carbon materials have great potential for development and play a non-negligible role in the research and application of Li-S batteries. Carbon nanomaterials with unique catalytic properties, which have few surface defects and can be doped, externally attached groups, and other methods to change the nature of carbon materials can be used to achieve the purpose of reducing defects [29]. To resolve the dissolution challenges of polysulfides, the surface functionalization of Gr significantly enhances the interaction with Li_2_S_8_/Li_2_S_4_ through the formation of ligand covalent Li-O bonds at the lithiation stage [21,24]. Due to the covalent nature of the Li-O bonds, the polysulfides are well retained inside the cathode, and the polysulfides also promote the interfacial charge transfer during the deposition of Li_2_S_8_ and Li_2_S_4_, which improves the conductivity of the electrodes. Moreover, MXene, an emerging two-dimensional nanomaterial, has garnered significant interest for its exceptional qualities, such as its hydrophilicity, remarkable electrical conductivity, stability, and outstanding electrochemical properties. These distinctive physical and chemical attributes render it highly valuable across a range of applications, encompassing electrocatalysis, supercapacitors, semiconductors, batteries, sensing technologies, biomedicine, water splitting, and photocatalysis [30,31,32,33,34,35]. MXenes holds great promise for the development of high-performance Li-S batteries. Their metallic conductivity facilitates rapid electron transport, ensuring the efficient utilization of sulfur and thus, high capacity in Li-S batteries. Moreover, the terminations on the surface MXene contribute to robust performance, effectively binding with Li_2_S_n_ to prevent the shuttle effect [10,11,12,16,26,29,36,37,38,39,40]. Furthermore, MXenes with various terminations demonstrate effective catalytic activity in converting Li_2_S_n_ to Li_2_S, thereby enhancing the kinetics of redox cycling [24,41,42,43]. The structural diversity of MXenes also offers significant potential for loading abundant amounts of sulfur or Li and accommodating volume expansion [44,45,46].

The dissolution phenomenon of polysulfides in Li-S batteries is essentially due to the high solubility of the solvents for polysulfides in commonly used electrolytes, so lower solubility solvents are sought. From previous research, 1,1,2-Trichloroethane and tetrahydrofuran can act as promising additives for Li-S batteries because of the high dielectric constant, as proposed by Meera et al. [47]. Li-S batteries are now believed to primarily use 1,2-dimethoxyethane (DME) and 1,3-dioxolane (DOL) in varying ratios because of their low viscosity, enhanced ionic conductivity, and high polysulfide solubility. However, multiple DME/DOL molecules may coordinate with the polysulfide in a homogeneous or heterogeneous way at the same time, changing their chemical composition and impacting battery performance. Because of its wide temperature range, excellent chemical and electrochemical stability, and efficient dissociation of Li ions, ionic liquid at room temperature is a useful solvent alternative [48]. Moreover, the addition of ionic liquid to the Li-S cell system accomplishes two special goals [49,50]: High viscosity retards polysulfide migration and reduces polysulfide dissolving due to a poor donor ability that is largely dependent on the anionic structure. Together, these help to reduce the shuttle phenomenon.

Nowadays, the intricate reaction process makes it challenging to represent the Li-S theoretical model both mechanistically and systemically. However, in this thesis, quantum chemical calculations were performed on MXene materials to explore their adsorption properties for polysulfides [51], at the same time, modified carbon material models were built to screen the material with good adsorption of small molecule sulfides, to improve the performance of the sulfur cathode and the service life and cyclability and stability of Li-S batteries. Moreover, we screened out ionic liquids with lower binding energy for use in electrolytes. Ultimately, we compared the binding affinity between mono-layer MXene and modified Gr, in which MXene drew a better performance. Also, we concluded five combinations of MXene and electrolytes to alleviate the dissolution of Li_2_S, Li_2_S_4_, Li_2_S_8_, Li_2_S_8_-2, and S_8_, respectively. This paper aims to describe one of the major challenges in the development of Li-S batteries-, namely the dissolution of polysulfides in the electrolyte solution, outline the interaction between Gr and sulfides, and inspire the exploration of the sulfur-fixing mechanism of modified carbon materials, which is the main direction of the research in this thesis. In this work, we proposed a pathway to alleviate the polysulfide dissolution in the Li-S batteries using quantum chemistry, in detail, the screening of appropriate combinations of cathode materials with electrolyte composition. This method results in reduced operation time compared to the experiment and guides use in practical situations.

## 2. Results and Discussion

### 2.1. Graphite Oxide Materials Frontier Molecular Orbital Analysis

**Figure 1:**
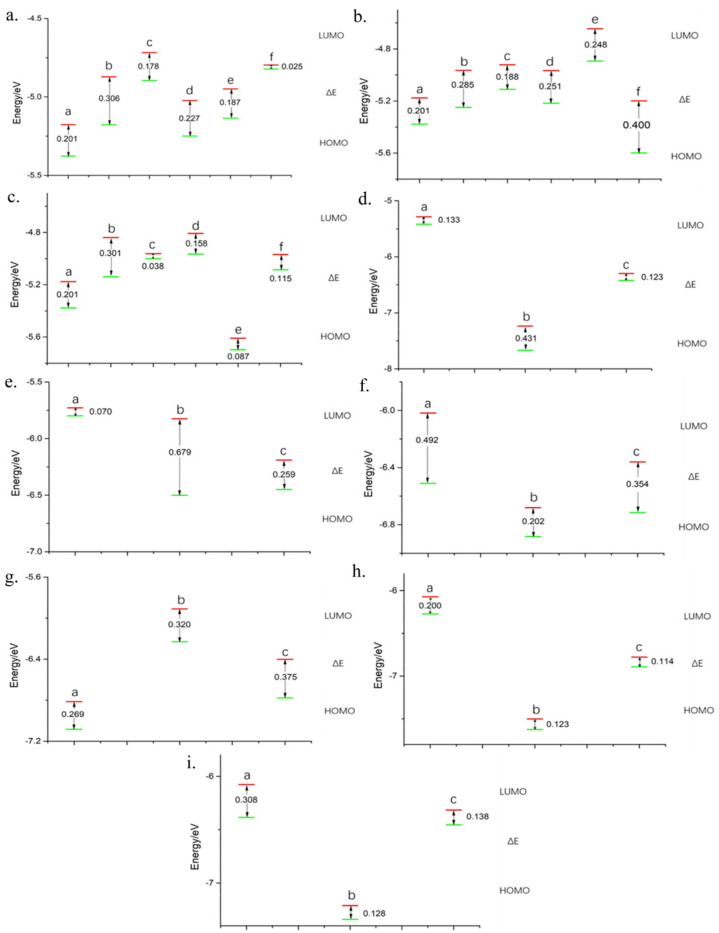
(**a**) Values of E_LUMO_, E_HOMO,_ and ΔE for each structure: (a) Gr (b) externally attached hydroxyl Gr (c) externally attached carboxyl Gr (d) externally attached bis-hydroxyl Gr (e) externally attached bis-carboxyl Gr (f) externally attached hydroxyl and carboxyl Gr. (**b**) Values of E_LUMO_, E_HOMO_ and ΔE for each structure: (a) Gr (b) externally attached hydroxyl Gr (c) externally attached carboxyl Gr (d) externally attached bis-hydroxyl Gr (e) externally attached bis-carboxyl Gr (f) externally attached hydroxyl and carboxyl Gr. (**c**) Values of E_LUMO_, E_HOMO_ and ΔE for each structure: (a) Gr (b) externally attached hydroxyl Gr (c) externally attached carboxyl Gr (d) externally attached bis-hydroxyl Gr (e) externally attached bis-carboxyl Gr (f) externally attached hydroxyl and carboxyl Gr. (**d**) Value of each structure E_LUMO_, E_HOMO_ with ΔE: (a) Py^3+^-BF_4_^−^ (b) Py^3+^-PF_6_^−^ (c) Py^3+^-TFSI^−^. (**e**) Value of each structure E_LUMO_, E_HOMO_ with ΔE: (a) P13^+^-BF_4_^−^ (b) P13^+^-PF_6_^−^ (c) P13^+^-TFSI^−^. (**f**) Values of structures E_LUMO_, E_HOMO_ with ΔE: (a) PP13^+^-BF_4_^−^ (b) PP13^+^-PF_6_^−^ (c) PP13^+^-TFSI^−^. (**g**) Values of structures E_LUMO_-E_HOMO_ and ΔE: (a) PMIM^+^-BF_4_^−^ (b) PMIM^+^-PF_6_^−^ (c) PMIM^+^-TFSI^−^. (**h**) Values of structures E_LUMO_, E_HOMO,_ and ΔE: (a) N1113^+^-BF_4_^−^ (b) N1113^+^-PF_6_^−^ (c) N1113^+^-TFSI^−^. (**i**) Value of each structure E_LUMO_, E_HOMO,_ and ΔE: (a) N3333^+^-BF_4_^−^ (b) N3333+-PF_6_^−^ (c) N3333^+^-TFSI^−^.

Building upon pristine Gr, oxygen-functional groups, encompassing hydroxyl and carboxyl moieties, were externally appended to the pyridine, pyrrole, and graphite sites of Gr. This resulted in the design of fifteen distinct Gr oxide configurations. The criteria for selecting promising candidates are elevated Highest Occupied Molecular Orbital (HOMO) energy values, lowered Lowest Unoccupied Molecular Orbital (LUMO) energy values, and reduced ΔE values. Figure 1a reveals that the HOMO energy values of Gr, bearing external oxygen-functional groups, have all seen an increase compared to the unmodified Gr structure. However, Gr structures with externally appended hydroxyl, bi-hydroxyl, and bi-carboxyl groups exhibit relatively modest changes in HOMO energy values. Conversely, Gr structures with externally attached hydroxyl and carboxyl groups demonstrate more significant alterations, resulting in higher energy levels. Notably, the vacant orbitals in these structures are inclined towards forming stronger adsorption bonds. When scrutinized in terms of ΔE, it becomes evident that Gr structures with externally attached hydroxyl and bi-hydroxyl groups exhibit elevated ΔE values in comparison to pristine Gr, potentially hindering electron transfer between HOMO and LUMO orbitals, and impeding robust adsorption. Conversely, Gr structures with externally appended carboxyl, bi-carboxyl, hydroxyl, and carboxyl groups demonstrate diminished ΔE values relative to unmodified Gr. Among these, Gr structures with externally attached carboxyl and hydroxyl groups exhibit lower LUMO values, signifying a heightened electron acceptance capacity, and facilitating faster electron transfer rates between HOMO and LUMO orbitals, thereby enhancing adsorption potential. In Figure 1b, except for Gr structures with externally attached hydroxyl and carboxyl groups, the remaining four Gr oxide structures exhibit augmented HOMO energy values compared to pristine Gr. Among these, the HOMO energy value of Gr with externally attached bi-carboxyl groups is the highest, indicating its superior electron-donating ability and propensity to form robust adsorption bonds with vacant orbitals. However, this structure also possesses the highest LUMO value among all configurations, implying a reduced electron acceptance capacity. As the corresponding ΔE value increases, decelerating electron transfer rates between HOMO and LUMO orbitals, thereby compromising strong adsorption. Additionally, the externally appended carboxylated Gr exhibits a decrease in ΔE value along with an increase in HOMO energy value, signifying enhanced adsorption stability and performance. In Figure 1c, externally attached hydroxyl Gr exhibits heightened HOMO and LUMO energy values, simultaneously, its ΔE value increases, indicating reduced adsorption stability. Conversely, externally attached carboxylic acid Gr, externally appended hydroxyl acid, and carboxylic acid Gr exhibit substantial reductions in ΔE values, indicating a predisposition towards stable adsorption and accelerated electron transfer rates, establishing these configurations as advantageous structures. Among these, externally attached hydroxyl and carboxyl Gr demonstrate the highest HOMO energy values, indicating significant advantages. By comprehensively comparing HOMO energy values, LUMO energy values, ΔE values, and other pertinent parameters across the fifteen Gr structures, five advantageous configurations have been identified: externally attached carboxylate at the pyridine site, externally appended hydroxyl and carboxyl Gr, externally attached carboxyl at pyrrole site Gr, externally appended bi-hydroxyl at graphite site, and externally attached hydroxyl and carboxyl Gr.

### 2.2. Ionic Liquid Frontier Molecular Orbital Analysis

Within this module, a systematic exploration was conducted holding the anionic species constant while varying the cationic components. Six distinct combinations were meticulously examined. The initial group encompassed Py^3+^ (pyridine) structures in tandem with anions BF_4_^−^, PF_6_^−^, and TFSI^−^. The second series introduced the cation P13^+^ (N-methyl-N-propylpyridine) combined with the same anions, BF_4_^−^, PF_6_^−^, and TFSI^−^. The third set featured ionic liquids comprising PP13^+^ (N-methyl-N-propylpiperidine) paired with anions PP13^+^-BF_4_^−^, PP13^+^-PF_6_^−^, and PP13^+^-TFSI^−^. The fourth collection highlighted the ionic liquid structure formed by the amalgamation of PMIM^+^ (1-methyl-3-propylimidazole) with anions BF_4_^−^, PF_6_^−^, and TFSI^−^. The fifth compilation showcased structures of N1113^+^ (trimethylpropyl quaternary ammonium) with corresponding anionic compositions, i.e., N1113^+^-BF_4_^−^, N1113^+^-PF_6_^−^, and N1113^+^-TFSI^−^. The sixth series constituted structures of an ionic liquid comprising the cation N3333^+^ (tetrapropyl quaternary ammonium), specifically, N3333^+^-BF_4_^−^, N3333^+^-PF_6_^−^, and N3333^+^-TFSI^−^.

As illustrated in Figure 1d, within the initial set, the ionic liquid structure formed by the combination of Py^3+^ and PF_6_^−^ exhibits the lowest HOMO energy value when juxtaposed with the other two structures. This indicates a diminished electron-donating capacity, rendering vacant orbitals less proficient in establishing robust adsorptive bonds. Moreover, it showcases the highest energy difference value, signifying a shortfall in adsorption stability and a challenge in achieving substantial adsorption. Consequently, Py^3+^-PF_6_^−^ is designated as the preferred ionic liquid structure. Within the second collection, P13^+^-PF_6_^−^ emerges as the prominent composition for similar reasons, where the energy value of the LUMO is notably elevated (Figure 1e). Upon evaluating in terms of the HOMO energy value, LUMO energy value, and ΔE value, it is evident that PP13^+^-PF_6_^−^ within the third compilation exhibits the lowest HOMO energy value, the lowest LUMO energy value, and the smallest ΔE value (Figure 1f), in contrast to screening requisites. In contrast, the combination of PP13^+^ and TFSI^−^ manifests entirely antithetical properties, indicating its suitability as the preferred structure. In the fourth set, PMIM^+^-BF_4_^−^ emerges as the optimal choice, potentially yielding modest binding energy when coupled with polysulphides, thereby augmenting the performance of Li-S battery electrolyte solutions (Figure 1g). Among the co-structures, the preferred parameter for consideration is the energy value of HOMO. Within the fifth collection, the N1113^+^-PF_6_^−^ structure manifests limited electron-donating capacity, rendering it an eligible ionic liquid structure (Figure 1h). Similar to the two aforementioned cation-composed structures, within the sixth compilation, the ionic liquid’s HOMO, LUMO energy value, and ΔE value for the N3333^+^-PF_6_^−^ composition rank lowest among the three structures (Figure 1i). Primarily assessed through the lens of the low HOMO energy value, it indicates a challenge in forming robust adsorption bonds, resulting in inadequate adsorption performance, thus rendering it an optimal ionic liquid structure. Through the systematic screening of 18 ionic liquid structures, a total of six structures have been identified: Py^3+^-PF_6_^−^, P13^+^-PF_6_^−^, PP13^+^-TFSI^−^, PMIM^+^-BF_4_^−^, N1113^+^-PF_6_^−^, and N3333^+^-PF_6_^−^. This selection is attributed to the inherent difficulty in forming strong adsorption bonds between vacant orbitals and ionic liquid structures, potentially leading to limited binding energy with polysulfides. Consequently, these six structures have been earmarked for subsequent stages of computational analysis.

### 2.3. Adsorption of Polysulfide with Graphite Oxide

**Figure 2:**
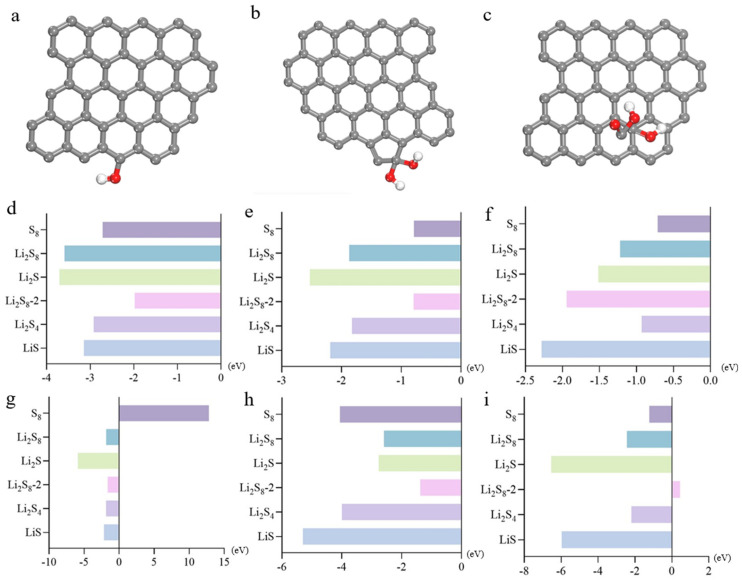
In the pictures, the gray atoms refer to carbon atoms, the red is oxygen, the white is hydrogen (**a**) Pyridine-site externally attached hydroxyl group Gr. (**b**) Pyrrole-site externally attached double hydroxyl group Gr. (**c**) Graphite-sites externally attached hydroxyl and carboxyl group Gr. (**d**) The binding energy of Gr and polysulfide. (**e**) The binding energy of Gr to polysulfides with externally attached carboxyl groups at the pyridine site. (**f**) Pyridine sites externally attached to hydroxyl and carboxyl groups Gr binding energy to polysulfides. (**g**) Pyrrole site externally attached to carboxyl group Gr binding energy to polysulfides. (**h**) Graphite sites externally attached to bis-hydroxy Gr with polysulfide binding energy. (**i**) Graphite sites externally attached to hydroxyl and carboxyl groups Gr binding energy to polysulfides.

In the pursuit of improved adsorption performance, graphene structures externally modified with oxygen-containing groups have been meticulously screened, i.e., graphite-site externally attached hydroxyl group and carboxyl group, pyridine-site externally attached hydroxyl group and carboxyl group, and pyrrole-site externally attached hydroxyl group and carboxyl group. The evaluation hinges on the binding energies of these graphite oxide electrode materials with lithium polysulfides. Elevated binding energies, relative to pristine graphene structures, signify superior adsorption performance for lithium polysulfides and hold the potential to ameliorate the solubility of polysulfides in electrolytes, a part of the theoretical model can be viewed in Figure 2a–c.

In Figure 2d, the binding energy between Gr and Li_2_S is notably most pronounced at −3.704 eV, indicating heightened adsorption on Li_2_S. This suggests that employing Gr material can effectively mitigate the dissolution of Li_2_S molecules in the electrolyte solution. The binding energies of Gr with polysulfides externally attached to the pyridine site are reduced, especially the two small molecules of Li_2_S_8_-2 and S_8_, which are −0.795 eV, and −0.789 eV, respectively (Figure 2e). In Figure 2f, when polysulfides interact with Gr-bearing hydroxyl and carboxyl groups at the pyridine position, Li_2_S_4_ and S_8_ molecules exhibit significantly diminished binding energies of −0.930 eV and −0.714 eV, respectively. A noteworthy increase in binding energy is observed for the Li_2_S molecule when interacting with Gr externally carboxylated at the pyrrole site, compared to pristine Gr (Figure 2g). In contrast, the S_8_ molecule exhibits a positive binding energy of 12.804 eV with this Gr oxide, indicating an unstable adsorption structure with minimal influence on S_8_ solubility. The binding energies of LiS, Li_2_S_4_, Li_2_S_8_, and Li_2_S_8_-2, experience a uniform reduction to some extent compared to pristine Gr, accentuating the phenomenon of sulfur dissolution. Figure 2h demonstrates that interactions between LiS, Li_2_S_4_, and S_8_ polysulfides with Gr featuring externally connected double hydroxyl groups result in increased binding energies. Noteworthy is the highest binding energy observed for LiS molecule with Gr oxide, registering at −5.301 eV. Conversely, interactions of Li_2_S, Li_2_S_8_, and Li_2_S_8_-2 polysulfides with this specific Gr oxide structure reveal a contrasting trend. Figure 2i reveals a distinct trend. Specifically, the binding energies of LiS and Li_2_S polysulfides with Gr sites externally appended with hydroxyl and carboxyl groups exhibit an augmentation relative to their interaction with pristine Gr. However, the binding energy of the Li_2_S_8_-2 molecule with this Gr oxide structure registers a positive value of 0.438 eV. This instability hinders the establishment of a robust binding interaction between Li_2_S_8_-2 and the Gr oxide. Conversely, the binding energies of Li_2_S_4_, Li_2_S_8_, and S_8_ polysulfides with Gr oxide exhibit a relative reduction.

**Figure 3:**
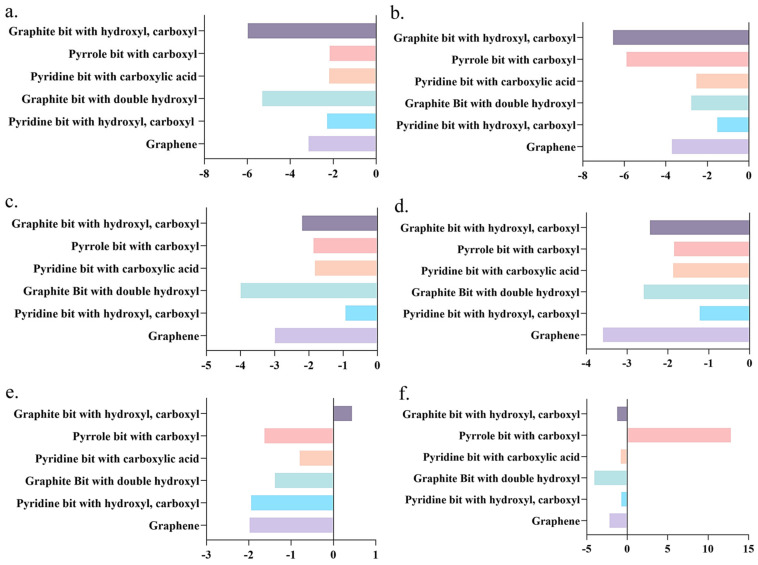
(**a**) LiS binding energy with Gr and Gr oxide. (**b**) Li_2_S binding energy to v and Gr oxide. (**c**) Li_2_S_4_ binding energy with Gr and Gr oxide. (**d**) The binding energy of Li_2_S_8_ with Gr and Gr oxide. (**e**) Li_2_S_8_-2 binding energy to Gr and Gr oxide. (**f**) S_8_ binding energy with Gr and Gr oxide.

In Figure 3a,b, Gr structures with double hydroxyl groups externally attached to graphite sites, and hydroxyl and carboxyl groups at the graphite site, exhibit notably higher binding energies with LiS and Li_2_S molecules, respectively. These findings underscore the superior adsorption capacity of these Gr structures for LiS and Li_2_S molecules, leading to a reduction of their solubility in the electrolyte solution. Among various Gr oxide structures, only the one featuring bis-hydroxy groups externally attached to graphite sites surpasses pristine Gr in binding energy with the Li_2_S_4_ molecule (−3.997 eV) (Figure 3c). Conversely, the Li_2_S_8_-2 molecule exhibits the highest binding energy with pristine Gr (Figure 3d), signifying superior adsorption. None of the investigated Gr oxide structures demonstrate an improvement in solubility for the Li_2_S_8_ molecule in the electrolyte solution (Figure 3e). This molecule exhibits the highest binding energy and optimal adsorption with pristine Gr (−3.590 eV). Moreover, the Gr oxide featuring a double hydroxyl group externally attached to graphite sites exhibits the highest binding energy and superior adsorption for the S_8_ molecule, surpassing pristine Gr. This suggests that the use of such Gr oxide can effectively mitigate the solubility of S_8_ in the electrolyte solution. However, the S_8_ molecule interacting with Gr externally carboxylated at the pyrrole site manifests a positive binding energy of 12.804 eV (Figure 3f), with similar ineffectiveness observed for other Gr oxide configurations.

### 2.4. Dissolution Behavior of Polysulfides in Electrolyte Solutions

**Figure 4:**
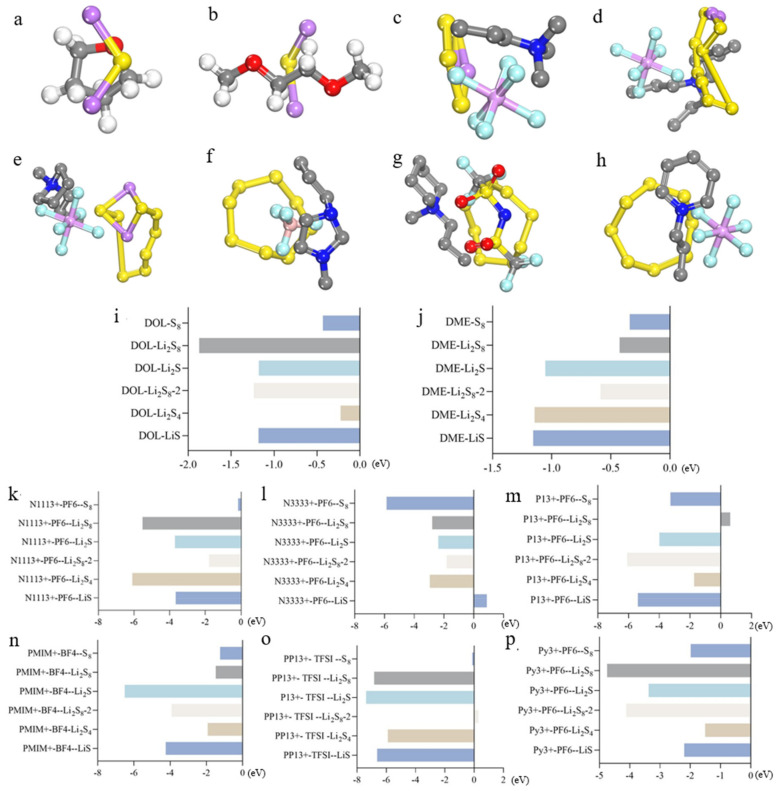
In the pictures, the gray atoms refer to carbon atoms, the red is oxygen, the white is hydrogen, the yellow one is sulfur, the blue one is nitrogen, the cyan-colored atom is fluoride, the pink atom is phosphorus, and the purple one is lithium. (**a**) DOL-LiS (**b**) DME-Li_2_S (**c**) N1113^+^-PF_6_^−^-Li_2_S_4_ (**d**) N3333^+^-PF_6_^−^-Li_2_S_8_ (**e**) P13^+^-PF_6_^−^-Li_2_S_8_-2 (**f**) PMIM^+^-BF_4_^−^-S_8_ (**g**) PP13^+^- TFSI^−^-S_8_ (**h**) Py^3+^-PF_6_^−^-S_8_ (**i**) The binding energy between DOL and polysulfides. (**j**) The binding energy between DME and polysulfides. (**k**) Ionic liquid N1113^+^-PF_6_^−^ binding energy with polysulfide. (**l**) Ionic liquid N3333^+^-PF_6_^−^ binding energy with polysulfide. (**m**) Ionic liquid P13^+^-PF_6_^−^ binding energy with polysulfide. (**n**) Ionic liquid PMIM^+^-BF_4_^−^ binding energy with polysulfide. (**o**) Ionic liquid PP13^+^-TFSI^−^ binding energy with polysulfide. (**p**) Ionic liquid Py^3+^-PF_6_^−^ binding energy with polysulfide.

Upon meticulous analysis of the data presented in Figure 4i, it becomes evident that DOL exhibits notably enhanced adsorption affinity towards LiS, Li_2_S, Li_2_S_8_, Li_2_S_8_-2, in comparison to Li_2_S_4_ and S_8_, where its performance is relatively weaker. Conversely, DME demonstrates superior adsorption towards LiS, Li_2_S, and Li_2_S_4_, but displays diminished adsorption capabilities towards Li_2_S_8_, Li_2_S_8_-2, and S_8_, (Figure 4j) a part of the theoretical model can be viewed in Figure 4a–h. Comparative analysis of DOL-adsorbed polysulfides (Figure 4i) and ionic liquid N1113^+^-PF_6_^−^-bound polysulfides reveals improved binding energies for most polysulfides, except S_8_, indicating reduced solubility of S_8_ in ionic liquids. For other polysulfides, heightened solubility in ionic liquids is observed. Similarly, comparing the binding energies of acquired ionic liquids with polysulfides to DME and polysulfides (Figure 4j) shows optimal results. Only LiS solubility is enhanced, while for others, solubility is exacerbated. Ionic liquid N1113^+^-PF_6_^−^ shows enhanced solubility exclusively for S_8_. When selecting lithium source electrode material, a higher concentration of S_8_ is advisable for improved Li-S battery performance with this ionic liquid (Figure 4k). The binding energies between the ionic liquids N33333^+^-PF_6_^−^ and polysulfides are listed in Figure 4l. It is evident that LiS exhibits a positive binding energy, indicating unstable adsorption with limited solubility. While other lithium polysulfides show higher solubility in ionic liquids compared to DOL (Figure 4i), this cannot fully address their susceptibility to dissolution. Compared with DME (Figure 4j), all binding energies of the obtained ionic liquids with polysulfides have increased, thus, such ionic liquids are counterproductive. The binding energy of ionic liquid P13^+^-PF_6_^−^ with the Li_2_S_8_ molecule is positive (0.614 eV), so by default, the adsorption structure is not stable (Figure 4m). The solubility of P13^+^-PF_6_^−^ to Li_2_S_8_ molecules is small, while the other adsorption structures have increased binding energies compared to the structure of DOL adsorbed polysulfides, especially the binding energy of adsorbed Li_2_S_8_-2 molecules is the largest, with the absolute value of 6.090 eV, which indicates that Li_2_S_8_-2 molecules dissolve in such electrolyte solutions with poor results. Compared with DME, the binding energy values of the adsorbed structures except Li_2_S_8_ molecules increased, and the five small-molecule polysulfides were more readily dissolved in ionic liquids P13^+^-PF_6_^−^. Therefore, the ionic liquid is suitable for lithium sources containing more Li_2_S_8_ molecules.

The binding energies of ionic liquid PMIM^+^-BF_4_^−^ and polysulphide Li_2_S_8_ molecules are reduced compared to that of DOL (Figure 4n), which indicates that Li_2_S_8_ molecules have a smaller solubility in these kinds of ionic liquids, and the binding energies of the other five small molecules of polysulphide are larger compared to DOL in the ionic liquids, especially Li_2_S molecules, which reaches absolute value of 6.508 eV. Similarly, compared with the binding energy of that the binding energies of ionic liquids and lithium polysulfide all increased, indicating that it cannot improve the phenomenon of the dissolution of polysulfide in the electrolyte solution. In summary, this ionic liquid reduces the solubility of Li_2_S_8_ molecules in the electrolyte solution but increases the solubility compared with DME, which is counterproductive. Ionic liquid PP13^+^-TFSI^−^ displays reduced solubility for Li_2_S_8_-2 and S_8_ molecules, making it suitable for Li-S batteries with higher concentrations of both molecules, leading to enhanced performance. However, the increased absolute values of binding energy calculations between the ionic liquid and six polysulfide molecules (Figure 4o) indicate the greater solubility of polysulfides in the ionic liquid compared to DOL or DME. This, however, fails to address the issue of polysulfide solubility, rendering this ionic liquid unsuitable for mitigating sulfur solubility phenomena. Furthermore, the binding energy calculations of Py^3+^-PF_6_^−^ and six polysulfide molecules, listed in Figure 4p, all indicate increased absolute values, demonstrating greater solubility of polysulfides in ionic liquid compared to DOL or DME. This, however, fails to address the issue of polysulfide solubility, rendering this ionic liquid unsuitable for mitigating sulfur solubility phenomena.

**Figure 5:**
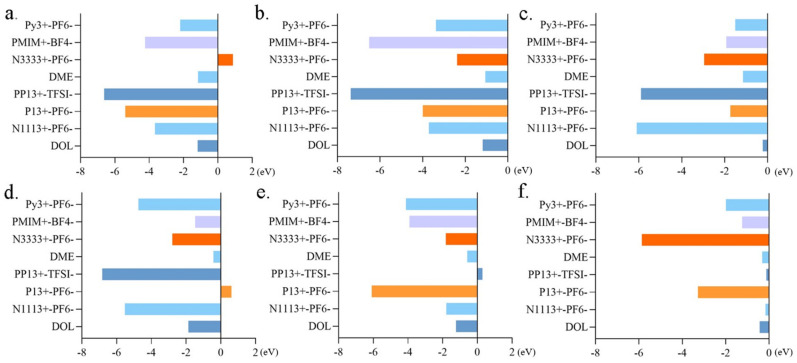
(**a**) The binding energy of LiS with ionic liquids and common solvents. (**b**) The binding energy of Li_2_S with ionic liquids and common solvents. (**c**) The binding energy of Li_2_S_4_ with ionic liquids and common solvents. (**d**) The binding energy of Li_2_S_8_ with ionic liquids and common solvents. (**e**) The binding energy of Li_2_S_8_-2 with ionic liquids and common solvents. (**f**) The binding energy of S_8_ with ionic liquids and common solvents.

In the case of the LiS molecule, a pronounced affinity was observed with Gr materials bearing external carboxyl and hydroxyl groups. Conversely, the binding energy with the ionic liquid N3333^+^-PF_6_^−^ manifested positivity, indicative of an unstable adsorption configuration, thereby impeding effective adsorption (Figure 5a). Pertaining to the Li_2_S molecule, the binding energy with all six tested ionic liquids surpassed values with DME, intimating that it may not be conducive to ameliorating the sulfur-solubilization phenomenon (Figure 5b). By the same token, the binding energies of Li_2_S_4_ molecules with common solvents, i.e., DOL, were surpassed by those with ionic liquids (Figure 5c). Among the surveyed ionic liquids, only P13^+^-PF_6_^−^ elicited a reduction in the binding energies of Li_2_S_8_ molecules relative to commonly employed solvents (Figure 5d). In the case of polysulfide Li_2_S_8_-2, its binding energy with the ionic liquid PP13^+^-TFSI^−^ ranked the lowest among the six assessed adsorption configurations, signifying diminished adsorption (Figure 5e). Consequently, an improvement in the sulfur-solubilization phenomenon is anticipated. The combination of S_8_ with the ionic liquids N1113^+^-PF_6_^−^ and PP13^+^-TFSI^−^ is anticipated to mitigate the solubility of S_8_ molecules in the electrolyte solution (Figure 5f).

### 2.5. Adsorption Behaviour of d-Ti_3_C_2_-MXene Monolayer for Polysulfide

**Figure 6:**
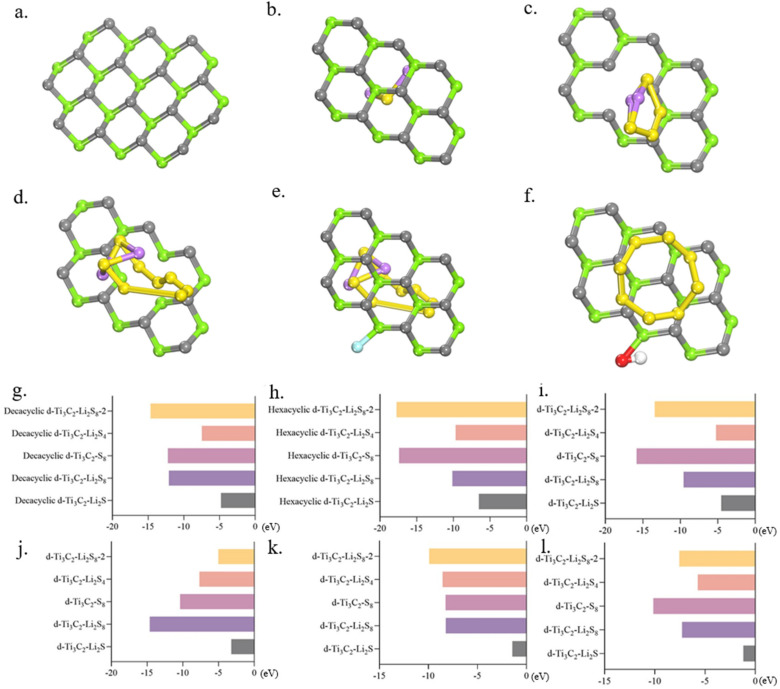
In the pictures, the gray atoms refer to carbon atoms, the red is oxygen, the white is hydrogen, the yellow one is sulfur, the cyan-colored atom is fluoride, the green atom is Ti, and the purple one is lithium. (**a**) Decacyclic d-Ti_3_C_2_ (**b**) Hexacyclic d-Ti_3_C_2_-Li_2_S (**c**) C-deficient hexacyclic d-Ti_3_C_2_-Li_2_S_4_ (**d**) Ti defective hexacyclic d-Ti_3_C_2_-Li_2_S_8_ (**e**) Externally attached F-atom hexacyclic d-Ti_3_C_2_-Li_2_S_8_-2 (**f**) Externally attached hydroxyhexacyclic d-Ti_3_C_2_-S_8_ (**g**) The binding energy of decacyclic d-Ti_3_C_2_ with polysulfide. (**h**) The binding energy of hexacyclic d-Ti_3_C_2_ with polysulfide. (**i**) The binding energy of hexacyclic d-Ti_3_C_2_ with unary C defect and polysulfide. (**j**) The binding energy of hexacyclic d-Ti_3_C_2_ with polysulfide in a single Ti defect. (**k**) The binding energy of external F atom hexacyclic d-Ti_3_C_2_ with polysulfide. (**l**) The binding energy of external hydroxyl hexacyclic d-Ti_3_C_2_ with polysulfide.

In order to analyze the better performance of alleviating the polysulfide dissolution phenomena in Li-S batteries, regarding the adsorption strength of polysulfide or Gr, we compared the computations of binding energies between MXene materials and polysulfide (Li_2_S, Li_2_S_4_, Li_2_S_8_, Li_2_S_8_-2, and S_8_) to screen the most appropriate path in this section. involving decacyclic d-Ti_3_C_2_ material, hexacyclic d-Ti_3_C_2_ material, defective d-Ti_3_C_2_ material, i.e., mono-C-deficient hexacyclic d-Ti_3_C_2_ and mono-C-deficient hexacyclic d-Ti_3_C_2_, moreover, heteroatom-modified d-Ti_3_C_2_ material, i.e., external F-atom hexacycles d-Ti_3_C_2_, and external hydroxy hexacyclic d-Ti_3_C_2_ with polysulfides yielded valuable insights, a part of the theoretical model are displayed in Figure 6a–f, Supplementary File will show more details.

Figure 6g displays original data elucidating the binding energies between Gr and polysulfides. Notably, the binding energy between decacyclic d-Ti_3_C_2_ and Li_2_S_8_-2 is notably pronounced at −14.659 eV. This underscores the efficacy of employing d-Ti_3_C_2_ MXene material in mitigating the dissolution of Li_2_S_8_-2 molecules within the electrolyte solution. The heightened adsorption affinity leads to a marked reduction in solubility within the electrolyte solution. Conversely, the binding energy between Gr and Li_2_S is considerably lower at −4.793 eV, indicating relatively suboptimal adsorption of Gr on Li_2_S_8_-2 molecules. Thus, decacyclic d-Ti_3_C_2_ is not preferable for inhibiting Li_2_S_8_-2 dissolution. The second-best and third-best adsorption affinity lies in S_8_ and Li_2_S_8_ with the binding energies of −12.240 eV and −12.092 eV, respectively. Through a meticulous evaluation of the binding energies, along with the selection of materials exhibiting heightened binding affinities, it is feasible to reduce the solubility of polysulfides within the electrolyte solution. Figure 6h illustrates augmented binding energies for hexacyclic d-Ti_3_C_2_ compared to the decacyclic ring, particularly for Li_2_S_8_-2 and S_8_, with values of −17.733 eV and −17.383 eV, respectively. As the aspect of other polysulfides, for Li_2_S, the binding energy value is −6.532 eV, the value is −9.691 eV for Li_2_S_4_, and −10.136 eV regarded as Li_2_S_8_. Noteworthy is that it exhibits the highest binding affinity among all MXene materials considered, signifying an exceptionally robust and stable adsorption interaction. Upon a comprehensive analysis of the binding energy data, consequently, the phenomenon of sulfur dissolution is exacerbated to the greatest extent, and hexacyclic d-Ti_3_C_2_ can act as the most optimal option among the selected MXene materials. However, the binding strength of Li_2_S_8_ is not as significant as other polysulfides.

Researching the effect of defect ring to tackle the sulfur dissolution puzzle, among all the adsorption energy data between unary C defect d-Ti_3_C_2_ and polysulfides, the d-Ti_3_C_2_-S_8_ has the greatest binding affinity (Figure 6i). Upon thorough analysis of the binding energy data, it is evident that for the Li_2_S_8_ molecule, the unary Ti defect d-Ti_3_C_2_ exhibits the strongest binding energy at −14.630 eV, indicating the most favorable adsorption between the two structures which have externally adopted functional groups (Figure 6j). With regard to the interaction between unary C defect d-Ti_3_C_2_ and S_8_, it has a value of −15.828 eV, which is less optimal than hexacyclic d-Ti_3_C_2_, but still a good performance. Conversely, the binding energies of the unary Ti defect d-Ti_3_C_2_ and the unary C defect d-Ti_3_C_2_ with the four polysulfides are lower than the former. This implies that, overall, d-Ti_3_C_2_ exhibits stronger adsorption properties for polysulfides, with defective d-Ti_3_C_2_ displaying poorer adsorption compared to pristine d-Ti_3_C_2_, and this observed decrease renders polysulfides more susceptible to dissolution in the electrolyte solution. However, upon interaction with heteroatom-modified hexacyclic d-Ti_3_C_2_, as depicted, there is a noticeable reduction in binding energies. This decrease renders polysulfides more susceptible to dissolution in the electrolyte solution. Particularly, Li_2_S and S_8_ molecules exhibit significantly diminished binding energies of −1.445 eV and −8.261 eV, respectively, indicating an increased likelihood of dissolution in the presence of an external F atom hexacyclic d-Ti_3_C_2_ in Figure 6k. This phenomenon, however, does not contribute to the enhancement of Li-S battery performance but rather exacerbates operational challenges. Furthermore, upon meticulous examination of the data in Figure 3d, a noteworthy decrease in binding energy is observed with the external hydroxyl group for the five types of polysulfides, indicating a diminished affinity for adsorption. The lowest binding energies of Li_2_S, Li_2_S_4_, Li_2_S_8_, and Li_2_S_8_-2 appear as the hydroxyl group is adopted (−1.194 eV, −5.727 eV, −7.299 eV, and −7.572 eV, respectively), which means that d-Ti_3_C_2_ with external hydroxyl group is the least likely choice for ameliorating the sulfur solubilization phenomenon (Figure 6l). After scrutinizing the binding energy values, it can be deduced that applying hexacyclic d-Ti_3_C_2_ is the most conducive to mitigation dissolution of Li_2_S, Li_2_S_4_, Li_2_S_8_-2, and S_8_, and unitary Ti-deficient hexacyclic d-Ti_3_C_2_ is most effective to adhere Li_2_S_8_ molecules.

**Figure 7:**
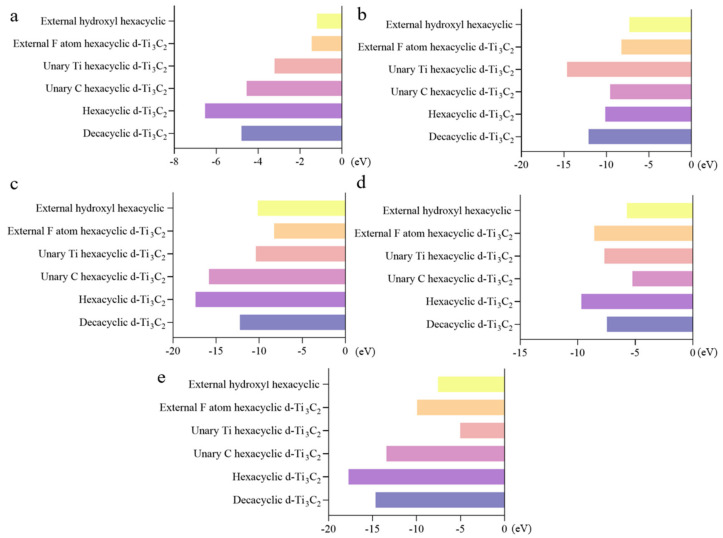
(**a**) The binding energy of Li_2_S with d-Ti_3_C_2_ MXene. (**b**) The binding energy of Li_2_S_8_ with d-Ti_3_C_2_ MXene. (**c**) The binding energy of S_8_ with d-Ti_3_C_2_ MXene. (**d**) The binding energy of Li_2_S_4_ with d-Ti_3_C_2_ MXene. (**e**) The binding energy of Li_2_S_8_-2 with d-Ti_3_C_2_ MXene.

The binding energies of MXene d-Ti_3_C_2_ with each polysulfide are higher than those of Gr with polysulfides, which indicates that d-Ti_3_C_2_ materials have a better adsorption performance for the five polysulfides, and it can reduce the dissolution of the polysulfides in the electrolyte solution to a large extent and improve the performance of the Li-S battery. Thus, summarizing all the calculated data above, an overall conclusion can be drawn. Pertaining to the Li_2_S molecule, the binding energy exhibited an increment when interfacing with hexacyclic d-Ti_3_C_2_. However, the binding energy of two kinds of ionic liquids surpassed these values, i.e., PMIM^+^-BF_4_^−^ and PP13^+^-TFSI^−^, intimating that it may not be conducive to ameliorating the sulfur-solubilization phenomenon (Figure 7a). Consequently, the conventionally employed solvent, DME, which has the lowest binding affinity among all the electrolytes was designated as the preferred electrolyte solution. The binding energies of Li_2_S_8_ molecules with unary Ti hexacyclic d-Ti_3_C_2_ are the highest. Among the surveyed ionic liquids, only P13^+^-PF_6_^−^ elicited a reduction in the binding energies of Li_2_S_8_ molecules relative to commonly employed solvents. Consequently, opting for unary Ti hexacyclic d-Ti_3_C_2_ as the electrode material, in conjunction with P13^+^-PF_6_^−^ ionic liquids, is conducive to curtailing the solubility of polysulfide Li_2_S_8_ in the electrolyte solution (Figure 7b). The binding energy of the S_8_ molecule with hexacyclic d-Ti_3_C_2_ was the highest, indicative of superior adsorption. Additionally, its binding energies with the ionic liquids N1113^+^-PF_6_^−^ and PP13^+^-TFSI^−^ were relatively diminished compared to commonly utilized solvents (Figure 7c). The adoption of such Gr materials and ionic liquids is anticipated to mitigate the solubility of S_8_ molecules in the electrolyte solution. In the case of the polysulfide Li_2_S_4_, hexacyclic d-Ti_3_C_2_ exhibited the most substantial binding energy. Conversely, the binding energies of Li_2_S_4_ molecules with commonly utilized solvents were surpassed by those with ionic liquids (Figure 7d). Thus, hexacyclic d-Ti_3_C_2_ is the recommended electrode material, with DOL identified as the preferred electrolyte solution. In the case of polysulfide Li_2_S_8_-2, the binding energy with the hexacyclic d-Ti_3_C_2_ was the most substantial, indicative of optimal adsorption. Conversely, its binding energy with the ionic liquid PP13^+^-TFSI^−^ ranked the lowest among the six assessed adsorption configurations, signifying diminished adsorption (Figure 7e). Consequently, an improvement in the sulfur-solubilization phenomenon is anticipated. To enhance the solubility of polysulfides within the electrolyte, a dual-pronged approach can be employed. Firstly, d-Ti_3_C_2_ MXene can be selected. Secondly, from an electrolyte perspective, the selection of ionic liquids with diminished binding energies with polysulfides can be pursued. Specifically, the screening criteria for binding energies were structured as follows, which were established to optimize the amelioration of dissolved sulfur and the reduction of polysulfide solubility within the electrolyte solution:

d-Ti_3_C_2_ MXene-LixSy > Gr oxide-LixSy > Gr-LixSy > DOL/DME-LixSy > Ionic liquids-LixSy

(Note: Comparisons of binding energies are comparisons of absolute values).

## 3. Conclusions

In this work, we summarized the appropriate combinations of the distinctive mono-layer cathode materials and electrolytes that we selected, which underscore the significant adsorption performance of the six-membered ring d-Ti_3_C_2_ MXene material, particularly in its interaction with five polysulfides. We delved into solving one of the major obstacles to the development of Li-S batteries—the dissolution of polysulphides in the electrolyte solution. In direct comparison to Gr, the d-Ti_3_C_2_ MXene exhibits notably stronger binding energies with individual polysulfides. Concerning the Li_2_S molecule, the strategic combination of hexacyclic d-Ti_3_C_2_ and DME emerges as a favorable approach. As for reducing the presence of Li_2_S_8_ molecules within the solution, utilizing P13^+^-PF_6_^−^ and unary Ti hexacyclic d-Ti_3_C_2_ demonstrates potential efficacy. Employing hexacyclic d-Ti_3_C_2_ as the electrode material alongside ionic liquids such as N1113^+^-PF_6_^−^ and PP13^+^-TFSI^−^ shows promise in diminishing the solubility of polysulfide S_8_ in the electrolyte solution. Notably, hexacyclic d-Ti_3_C_2_ displays substantial affinity with the polysulfide Li_2_S_4_. Furthermore, regarding reducing the solubilization of polysulfide Li_2_S_8_-2, the utilization of hexacyclic d-Ti_3_C_2_ in conjunction with PP13^+^-TFSI^−^ demonstrates notable efficacy. Within this investigation, we proposed a quantum chemistry-oriented methodology aimed at alleviating polysulfide dissolution in Li-S batteries. Specifically, our approach involves the screening of compatible combinations of cathode materials and electrolyte compositions. In contrast to experimental methodologies, this proposed approach demonstrates reduced operational time and holds the potential for practical application in real-world settings.

## Data Availability

Data generated or analyzed during this study are provided in fullwithin the article.

## Supplementary Materials

The following supporting information can be downloaded at: https://www.mdpi.com/article/10.3390/molecules29010002/s1, Figures S1–S24 and Tables S1–S37.
