# Supplementary material for: Enhancing Lithium-Sulfur Battery Performance by MXene, Graphene, and Ionic Liquids: A DFT Investigation
Source: Molecules. 2023 Dec 19;29(1):2. doi: 10.3390/molecules29010002 (PMC10779824; doi:10.3390/molecules29010002)
Supplement: Supplementary file 1 [file molecules-29-00002-s001.zip › molecules-2741893-supplementary.pdf]

Enhancing Lithium-Sulfur Battery Performance by MXene, Graphene, and Ionic Liquids: A DFT Investigation

Jianghui Cao [a], Sensen Xue [a], Jian Zhang [a], Xuefeng Ren [b,*], Liguo Gao [a], Tingli Ma [c, d, *], Anmin Liu [a,*]

[a] State Key Laboratory of Fine Chemicals, School of Chemical Engineering, Dalian University of Technology, China.

E-mail: liuanmin@dlut.edu.cn

[d] School of Ocean Science and Technology, Dalian University of Technology, Panjin, 124221, China.

E-mail: renxuefeng@dlut.edu.cn

[c] Department of Materials Science and Engineering, China Jiliang University, Hangzhou, 310018, China.

[d] Graduate School of Life Science and Systems Engineering, Kyushu Institute of Technology, 2-4 Hibikino, Wakamatsu, Kitakyushu, Fukuoka 808-0196, Japan.

E-mail: tinglima@life.kyutech.ac.jp

With the help of quantum chemical calculations, the properties of d-Ti_3C_2, the charge distribution of graphene oxide, molecular orbital energies, and the related properties of electrolyte solutions after the addition of ionic liquids were calculated by using the Dmol3 function of Materials Studio software, with a view to determine the reactivity and adsorption stability of the d-Ti_3C_2, graphene oxide, as well as the solubility of the electrolyte solution for polysulphides, sulfide solubility, and to screen out advantageous electrode materials and ionic liquids. The number of iterations and the number of self-consistent cycles was set to 1000. If they do not converge within 1000, the value can be increased. Moreover, the temperature is constant, i.e., 298.15K.

A total of 15 graphene oxide structures were designed on the basis of pristine graphene, and the structures were first processed for structural optimisation, then the energy calculations were completed in order to determine their reactivity and adsorption sites.

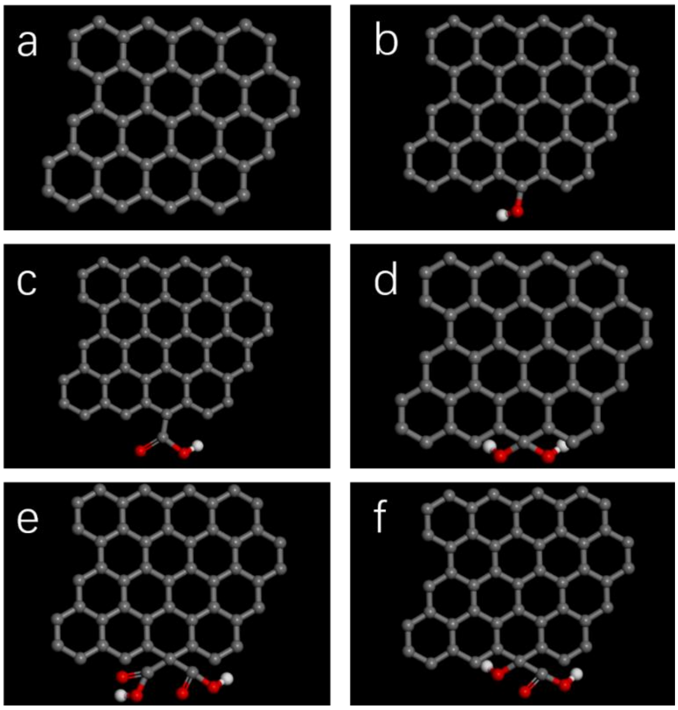

Figure S1 Schematic diagram of the molecular structure graphene and pyridine-site externally attached functional group: (a) Gr (b) externally attached hydroxyl group Gr (c) externally attached carboxyl group Gr (d) externally attached double hydroxyl group Gr (e) externally attached double carboxyl group Gr (f) externally attached hydroxyl and carboxyl group Gr

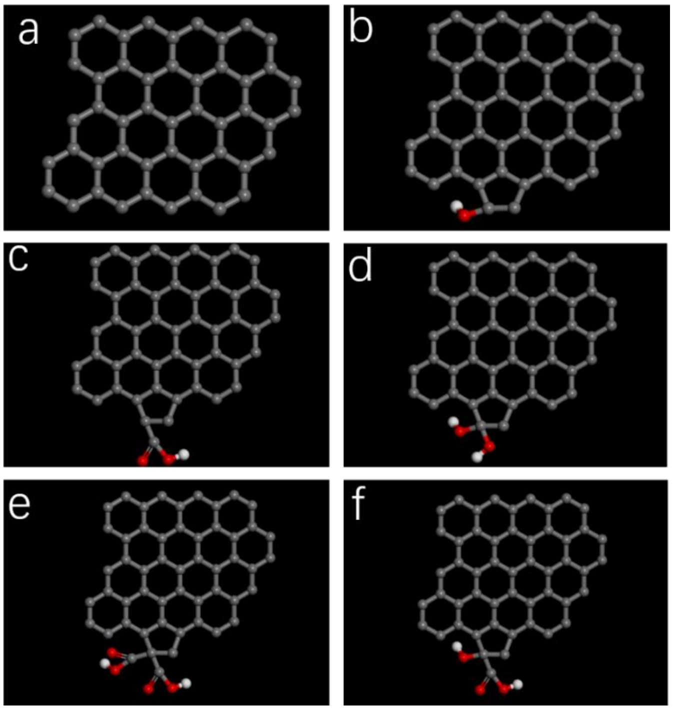

Figure S2 Schematic diagram of the molecular structure of graphene structures and five graphene oxide structures with oxygen-containing groups externally attached to the pyrrole site: (a) Gr (b) externally attached hydroxyl group Gr (c) externally attached carboxyl group Gr (d) externally attached double hydroxyl group Gr (e) externally attached double carboxyl group Gr (f) externally attached hydroxyl and carboxyl group Gr

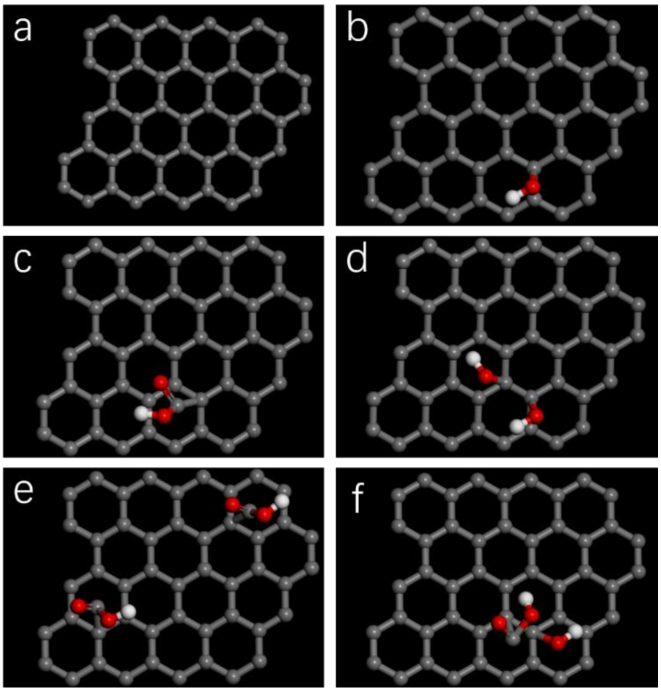

Figure S3 Schematic diagram of the molecular structure of graphene structures and five Gr structures with oxygen-containing groups externally attached to graphite sites: (a) Gr (b) externally attached hydroxyl group Gr (c) externally attached carboxyl group Gr (d) externally attached double hydroxyl group Gr (e) externally attached double carboxyl group Gr (f) externally attached hydroxyl and carboxyl group Gr

The calculation of binding energy in the research of behavioral studies on the adsorption of polysulphides by graphite oxide electrode materials encompasses three primary components: the total energy of the graphite oxide electrode material coupled with lithium polysulphide, the standalone total energy of the graphite oxide electrode material, and the standalone total energy of lithium polysulphide. This is mathematically represented as:

$$E = E_{\mathrm{Gr/Li}_x\mathrm{S}_y} - E_{\mathrm{Gr}} - E_{\mathrm{Li}_x\mathrm{S}_y}$$

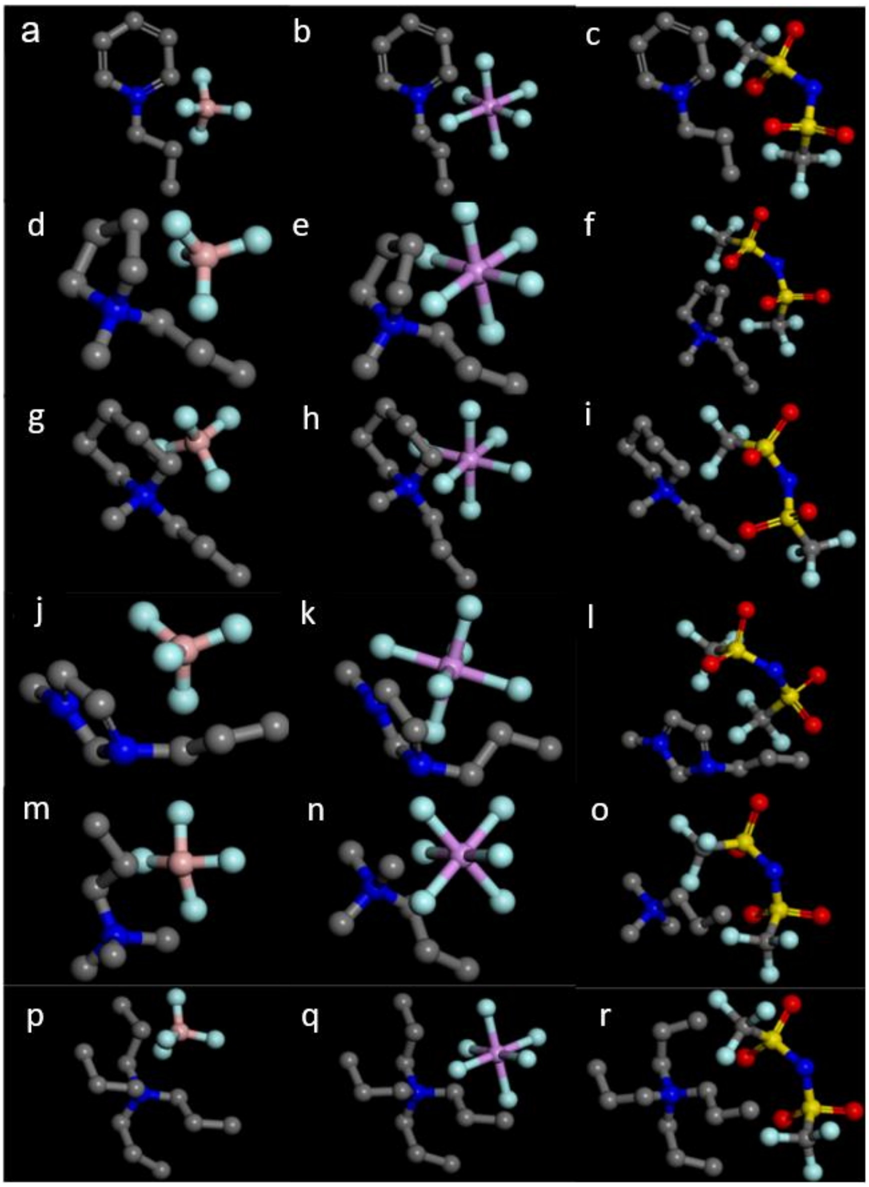

Figure S4 Schematic diagram of ionic liquid structure (a) Py^{3+}-BF_4^- (b) Py^{3+}-PF_6^- (c) Py^{3+}-$TFSI^-$ (d) $P13^+$-BF_4^- (e) $P13^+$-PF_6^- (f) $P13^+$-$TFSI^-$ (g) $PP13^+$-BF_4^- (h) $PP13^+$-PF_6^- (i) $PP13^+$-$TFSI^-$ (j) $PMIM^+$-BF_4^- (k) $PMIM^+$-PF_6^- (l) $PMIM^+$-$TFSI^-$ (m) $N1113^+$-BF_4^- (n) $N1113^+$-PF_6^- (o) $N1113^+$-$TFSI^-$ (p) $N3333^+$-BF_4^- (q) N3333+-PF_6^- (r) $N3333^+$-$TFSI^-$.

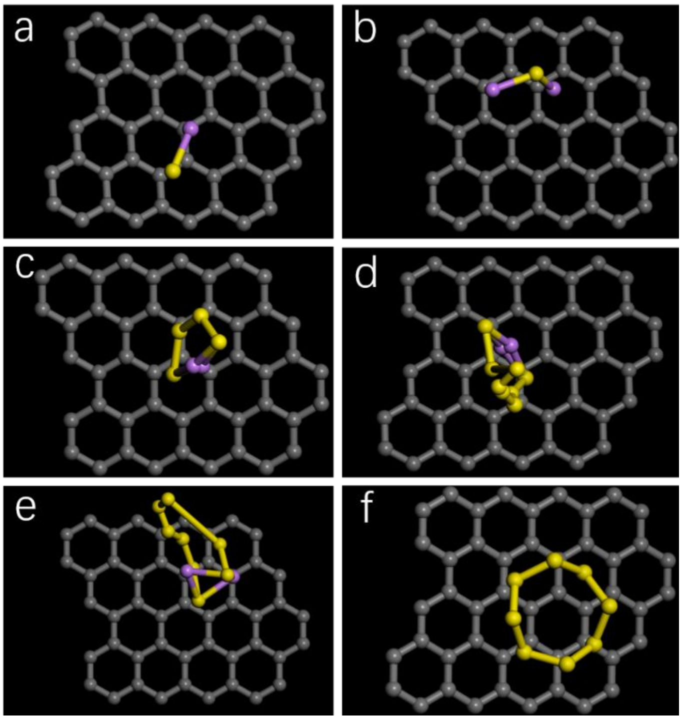

Figure S5 (a) Gr-LiS（b）Gr-Li_2S（c）Gr-Li_2S_4（d）Gr-Li_2S_8（e）Gr-Li_2S_8-2（f）Gr-S_8

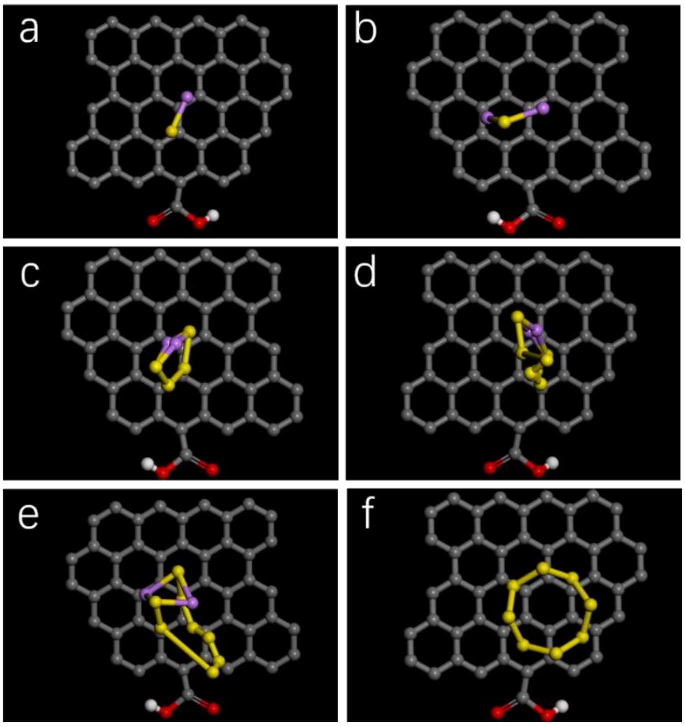

Figure S6 (a) Pyridine site externally attached carboxylate Gr-LiS (b) Pyridine site externally attached carboxylate Gr-Li_2S (c) Pyridine site externally attached carboxylate Gr-Li_2S_4 (d) Pyridine site externally attached carboxylate Gr-Li_2S_8 (e) Pyridine site externally attached carboxylate Gr-Li_2S_8-2 (f) Pyridine site externally attached carboxylate Gr-S_8

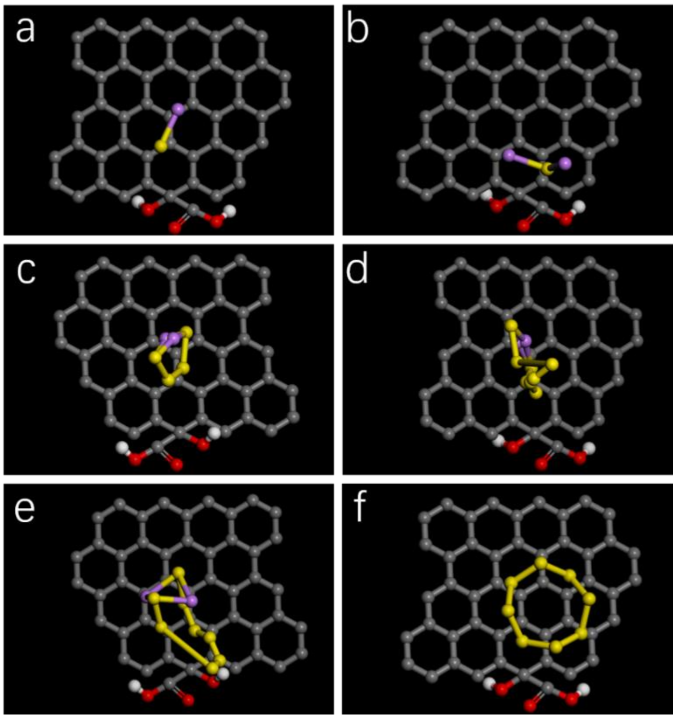

Figure S7 Schematic diagrams of the molecular structures of: (a) hydroxyl and carboxyl groups externally attached to the pyridine site Gr-LiS (b) hydroxyl and carboxyl groups externally attached to the pyridine site Gr-Li_2S (c) hydroxyl and carboxyl groups externally attached to the pyridine site Gr-Li_2S_4 (d) hydroxyl and carboxyl groups externally attached to the pyridine site Gr-Li_2S_8 (e) hydroxyl and carboxyl groups externally attached to the pyridine site Gr-Li_2S_8-2 (f) hydroxyl and carboxyl groups externally attached to the pyridine site Gr-S_8

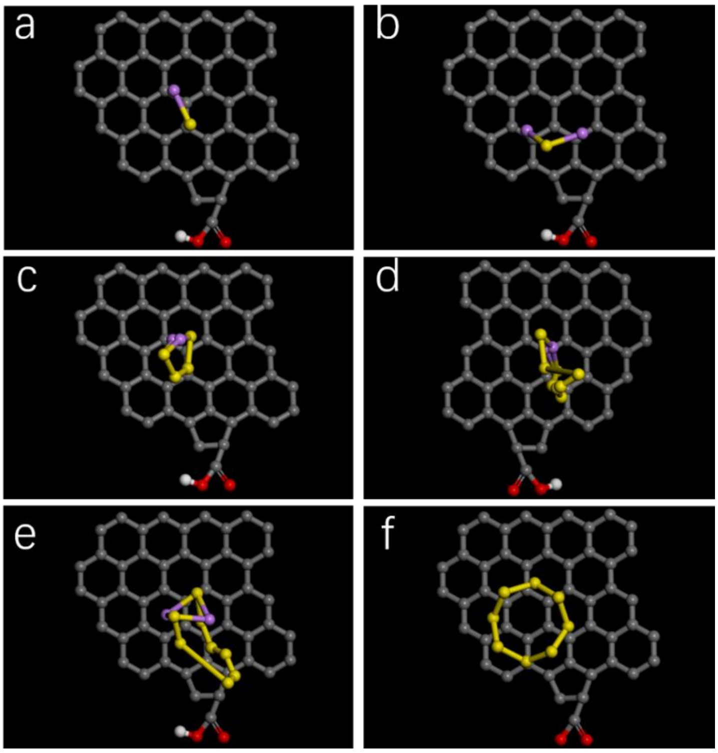

Figure S8 Schematic diagram of the molecular structures of: (a) pyrrole externally attached carboxylate Gr-LiS (b) pyrrole externally attached carboxylate Gr-Li_2S (c) pyrrole externally attached carboxylate Gr-Li_2S_4 (d) pyrrole externally attached carboxylate Gr-Li_2S_8 (e) pyrrole externally attached carboxylate Gr-Li_2S_8-2 (f) pyrrole externally attached carboxylate Gr-S_8

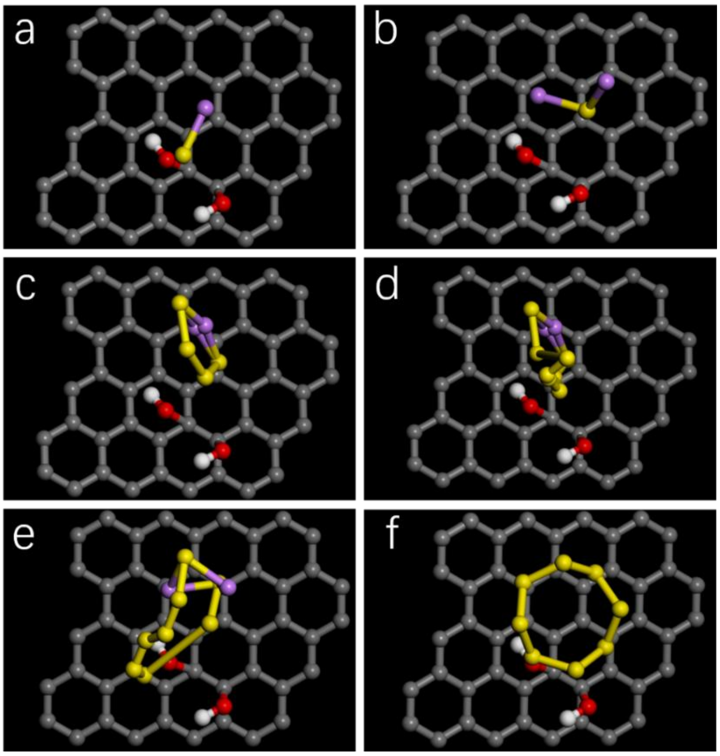

Figure S9 Schematic diagram of the molecular structures of: (a) graphite site externally attached bis-hydroxy Gr-LiS (b) graphite site externally attached bis-hydroxy Gr-Li_2S (c) graphite site externally attached bis-hydroxy Gr-Li_2S_4 (d) graphite site externally attached bis-hydroxy Gr-Li_2S_8 (e) graphite site externally attached bis-hydroxy Gr-Li_2S_8-2 (f) graphite site externally attached bis-hydroxy Gr-S_8

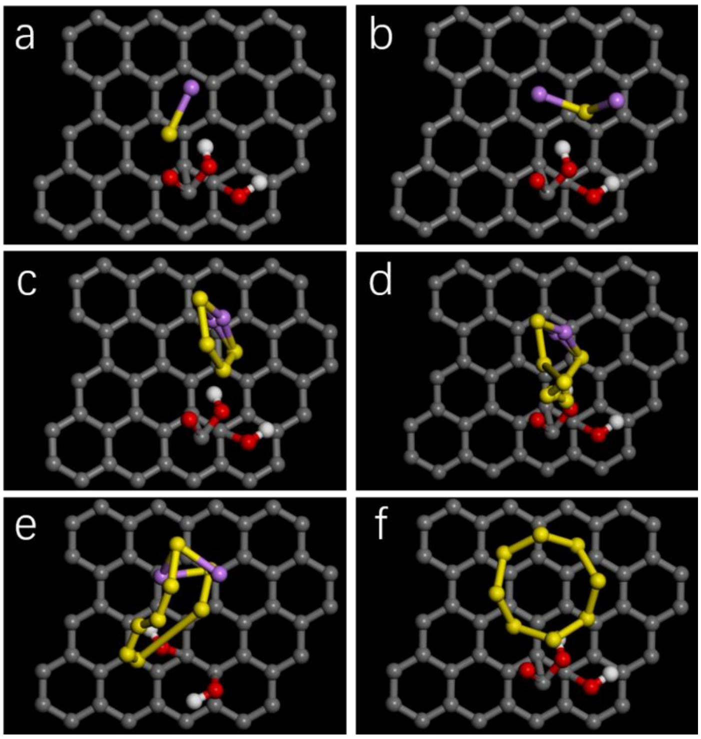

Figure S10 Schematic diagrams of the molecular structures of: (a) graphite site externally attached hydroxyl group and carboxyl group Gr-LiS (b) graphite site externally attached hydroxyl group and carboxyl group Gr-Li_2S (c) graphite site externally attached hydroxyl group and carboxyl group Gr-Li_2S_4 (d) graphite site externally attached hydroxyl group and carboxyl group Gr-Li_2S_8 (e) graphite site externally attached hydroxyl group and carboxyl group Gr-Li_2S_8-2 (f) graphite site externally attached hydroxyl group and carboxyl group Gr-S_8

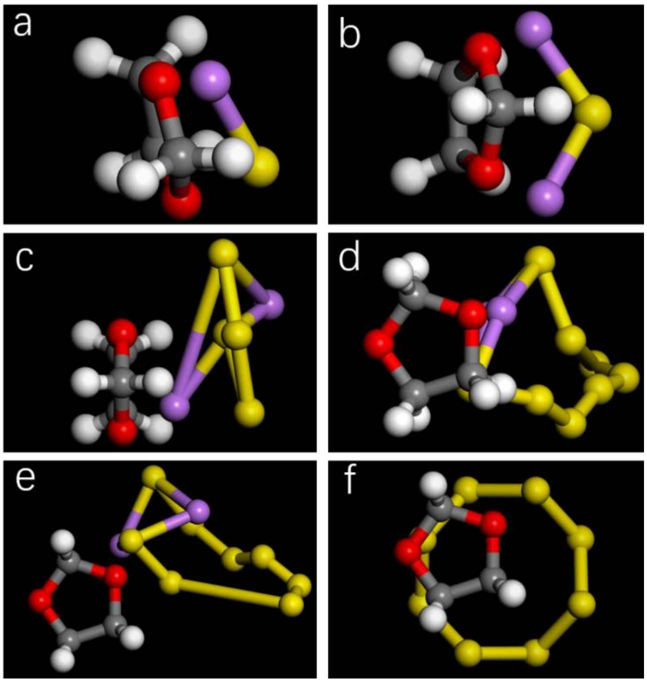

Figure S11（a）DOL-LiS（b）DOL-Li_2S（c）DOL-Li_2S_4（d）DOL-Li_2S_8（e）DOL-Li_2S_8-2（f）DOL-S_8

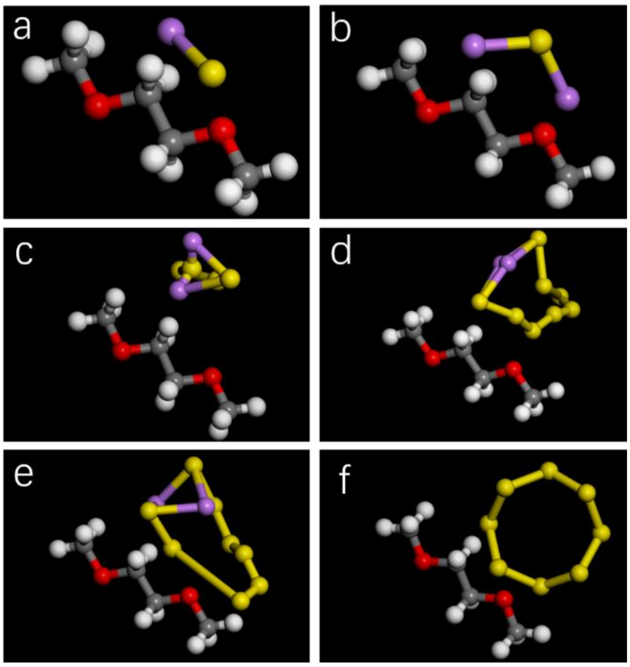

Figure S12（a）DME-LiS（b）DME-Li_2S（c）DME-Li_2S_4（d）DME-Li_2S_8（e）DME-Li_2S_8-2（f）DME-S_8

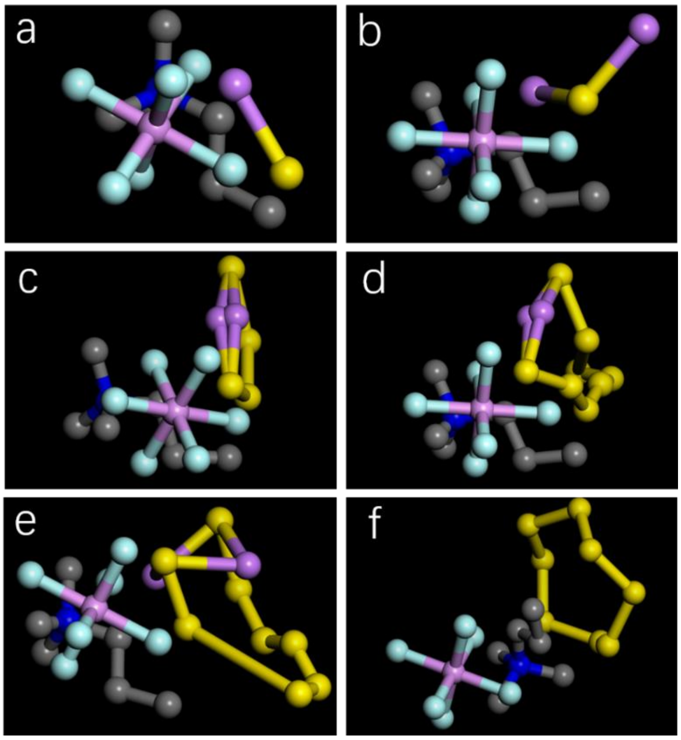

Figure S13 Schematic structure of ionic liquid adsorbed lithium polysulphide molecules: (a) $N1113^{+}$-PF_6^{-}-LiS (b) $N1113^{+}$-PF_6^{-}-Li2S (c) $N1113^{+}$-PF_6^{-}-Li_2S_4 (d) $N1113^{+}$-PF_6^{-}-Li_2S_8 (e) $N1113^{+}$-PF_6^{-}-Li_2S_8-2 (f) $N1113^{+}$-PF_6^{-}-S_8

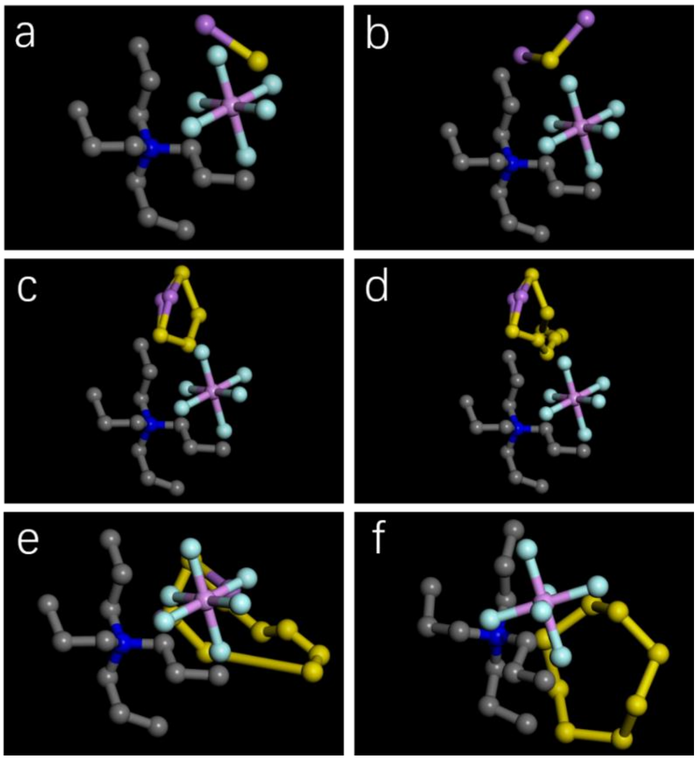

Figure S14 （a）$N3333^+$-PF_6^--LiS（b）$N3333^+$-PF_6^--Li_2S（c）$N3333^+$-PF_6^--Li_2S_4（d）$N3333^+$-PF_6^--Li_2S_8（e）$N3333^+$-PF_6^--Li_2S_8-2（f）$N3333^+$-PF_6^--S_8

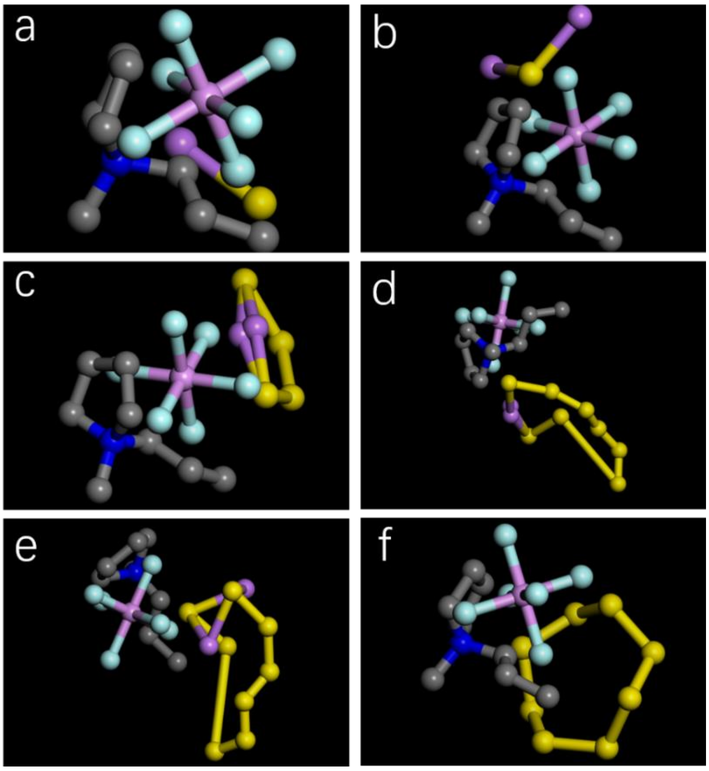

Figure S15 （a）$P13^+$-PF_6^--LiS（b）$P13^+$-PF_6^--Li_2S（c）$P13^+$-PF_6^--Li_2S_4（d）$P13^+$-PF_6^--Li_2S_8

（e）P13^{+}-PF$_6^{-}$-Li$_2$S$_8$-2（f）P13^{+}-PF$_6^{-}$-S$_8$

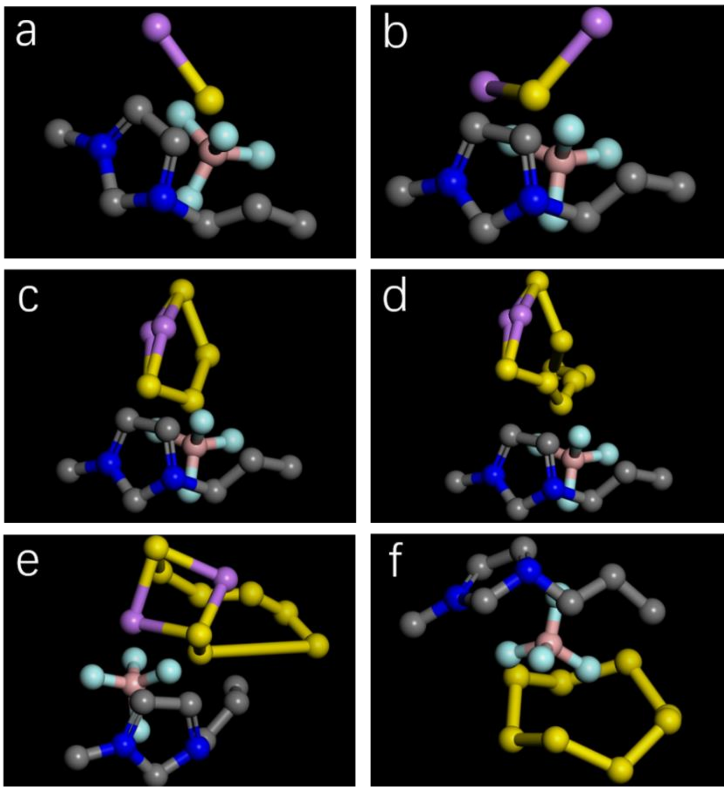

Figure S16 （a）PMIM^{+}-BF$_4^{-}$-LiS（b）PMIM^{+}-BF$_4^{-}$-Li$_2$S（c）PMIM^{+}-BF$_4^{-}$-Li$_2$S$_4$（d）PMIM^{+}-BF$_4^{-}$-Li$_2$S$_8$（e）PMIM^{+}-BF$_4^{-}$-Li$_2$S$_8$-2（f）PMIM^{+}-BF$_4^{-}$-S$_8$

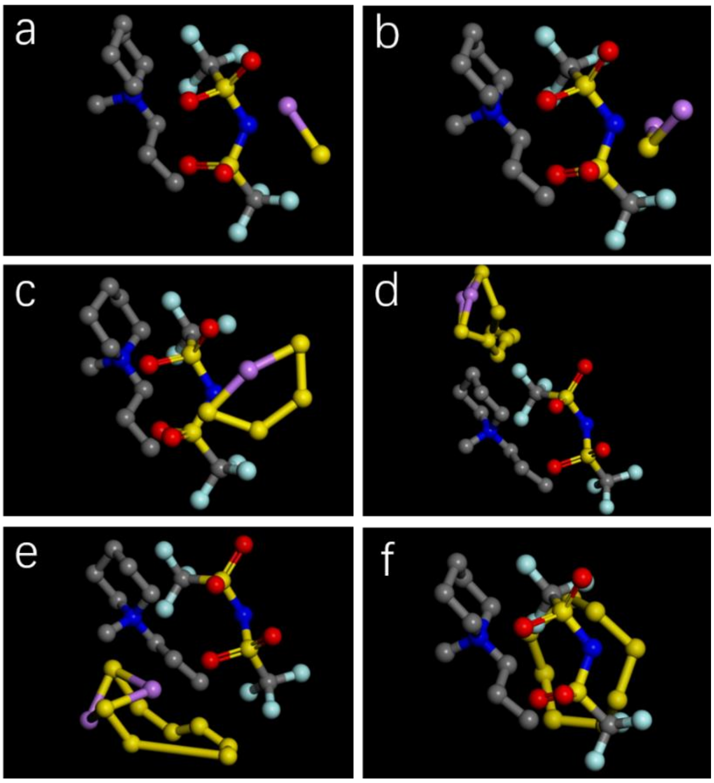

Figure S17 （a）$PP13^{+}$-$TFSI^{-}$-LiS（b）$PP13^{+}$- $TFSI^{-}$-Li_2S（c）$PP13^{+}$- $TFSI^{-}$-Li_2S_4（d）$PP13^{+}$- $TFSI^{-}$-Li_2S_8（e）$PP13^{+}$- $TFSI^{-}$-Li_2S_8-2（f）$PP13^{+}$- $TFSI^{-}$-S_8

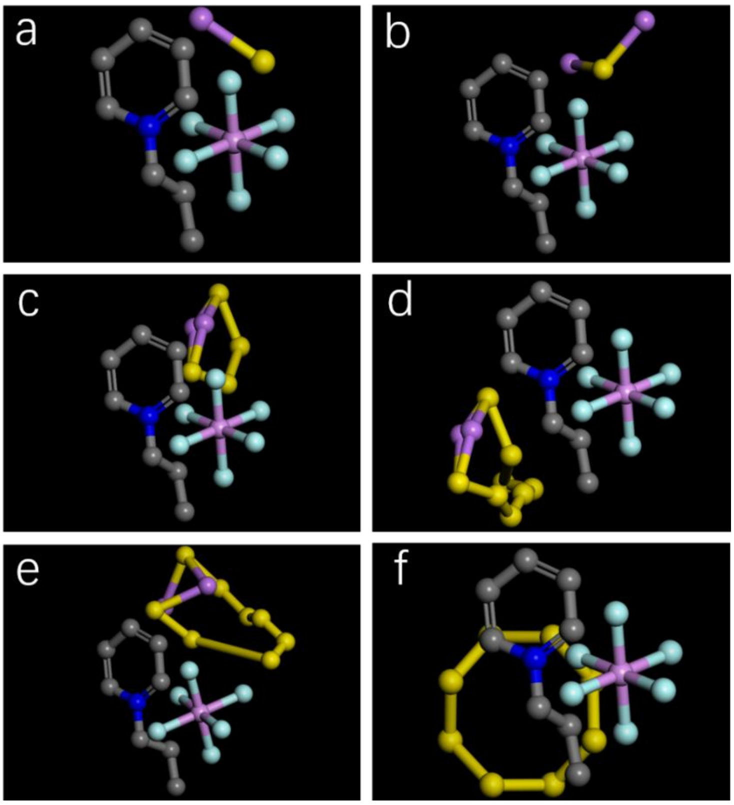

Figure S18 （a）Py^{3+}-PF_6^--LiS（b）Py^{3+}-PF_6^--Li_2S（c）Py^{3+}-PF_6^--Li_2S_4（d）Py^{3+}-PF_6^--Li_2S_8（e）Py^{3+}-PF_6^--Li_2S_8-2（f）Py^{3+}-PF_6^--S_8

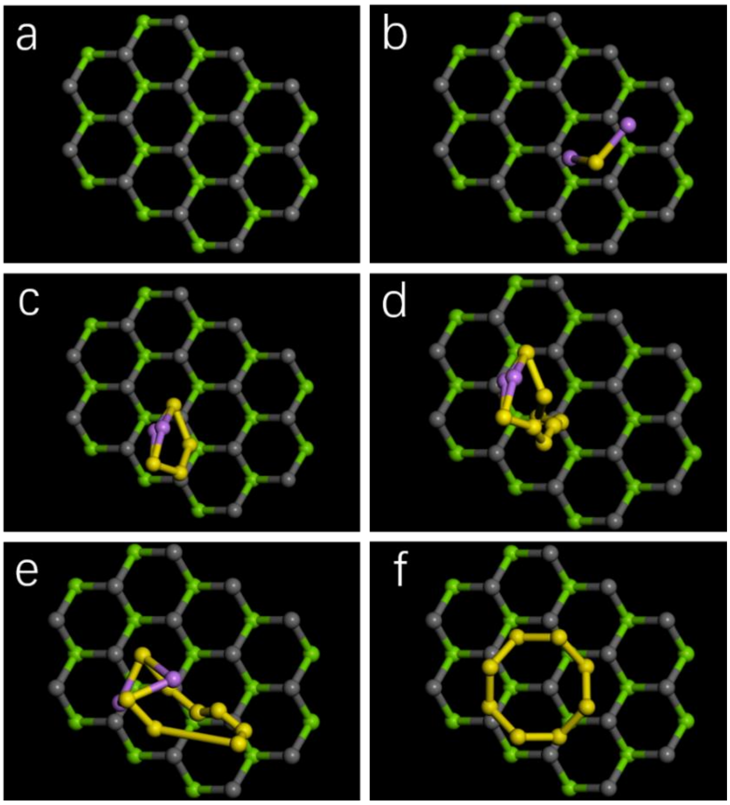

Figure S19 Schematic structure of decacyclic d-Ti_3C_2 and its adsorbed lithium polysulfide molecules: （a）d-Ti_3C_2（b）d-Ti_3C_2-Li_2S（c）d-Ti_3C_2-Li_2S_4（d）d-Ti_3C_2-Li_2S_8（e）d-Ti_3C_2-Li_2S_8-2（f）d-Ti_3C_2-S_8

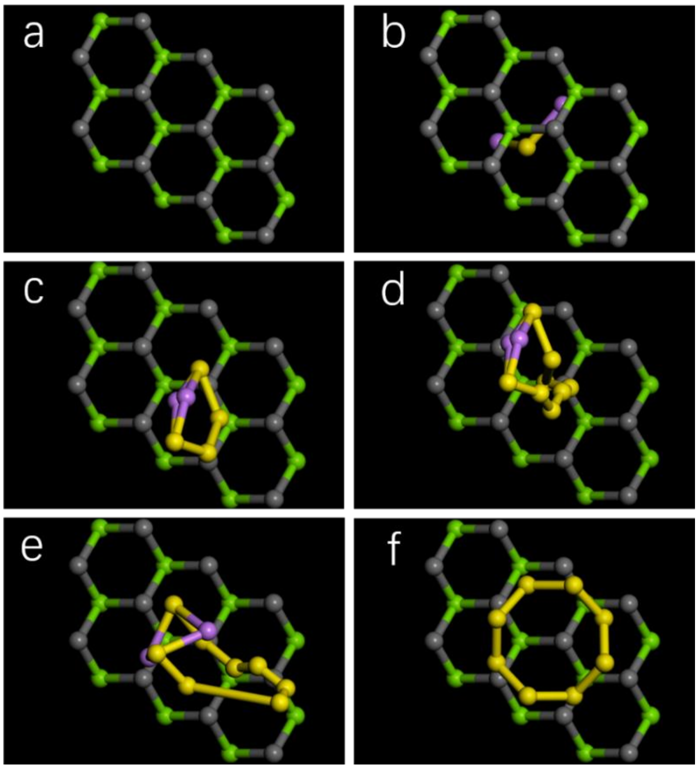

Figure S20 Schematic representation of the molecular structure of hexacyclic d-Ti_3C_2 and its adsorbed lithium polysulfide: （a）d-Ti_3C_2（b）d-Ti_3C_2-Li_2S（c）d-Ti_3C_2-Li_2S_4（d）d-Ti_3C_2-Li_2S_8（e）d-Ti_3C_2-Li_2S_8-2（f）d-Ti_3C_2-S_8

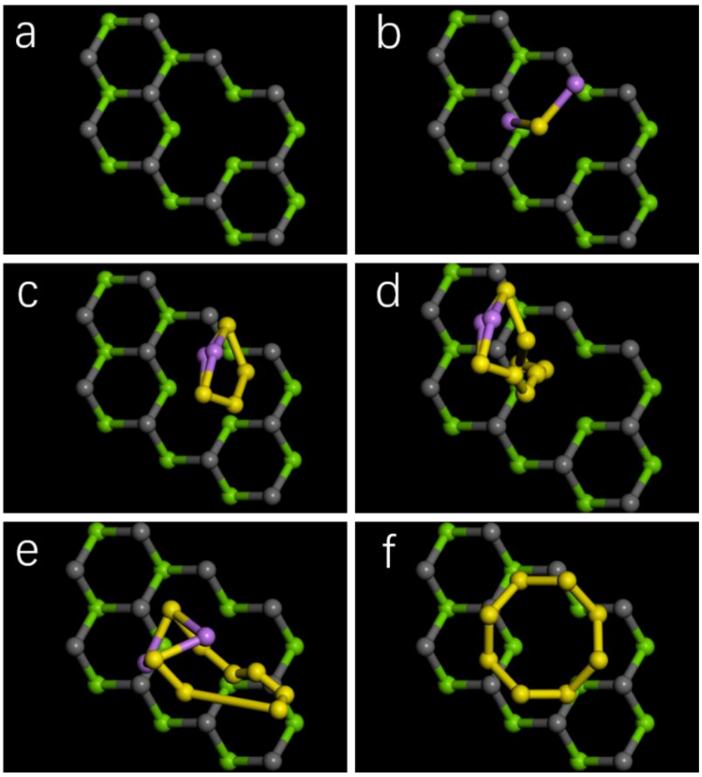

Figure S21 Schematic representation of the molecular structure of monomer C-deficient hexacyclic d-Ti_3C_2 and its adsorbed lithium polysulfide: （a）d-Ti_3C_2（b）d-Ti_3C_2-Li_2S（c）d-Ti_3C_2-Li_2S_4（d）d-Ti_3C_2-Li_2S_8（e）d-Ti_3C_2-Li_2S_8-2（f）d-Ti_3C_2-S_8

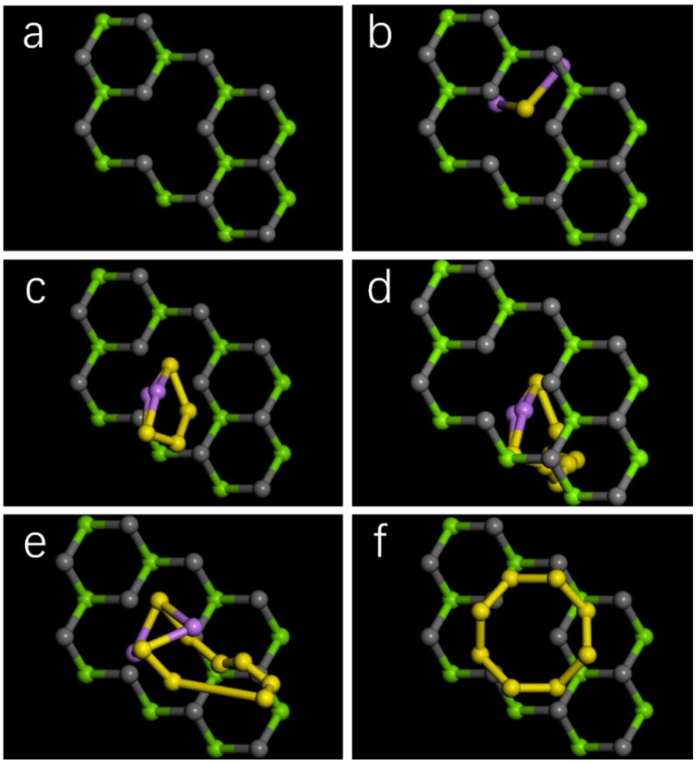

Figure S22 Schematic representation of the molecular structure of mono Ti defective hexacyclic d-Ti_3C_2 and its adsorbed lithium polysulphide: （a）d-Ti_3C_2（b）d-Ti_3C_2-Li_2S（c）d-Ti_3C_2-Li_2S_4（d）d-Ti_3C_2-Li_2S_8（e）d-Ti_3C_2-Li_2S_8-2（f）d-Ti_3C_2-S_8

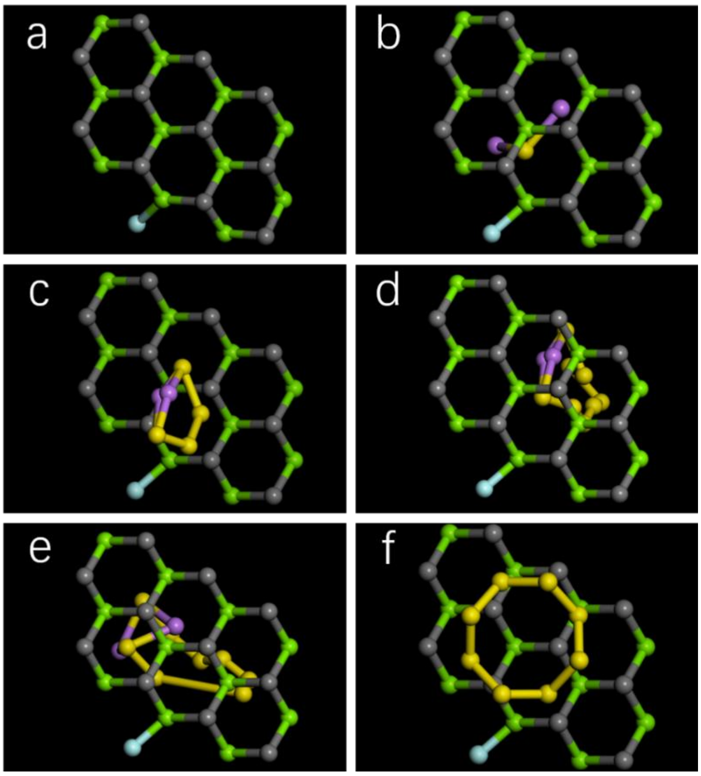

Figure S23 Schematic representation of the molecular structure of externally attached F-atom hexacyclic d-Ti_3C_2 and its adsorbed lithium polysulfide: （a）d-Ti_3C_2（b）d-Ti_3C_2-Li_2S（c）d-Ti_3C_2-Li_2S_4（d）d-Ti_3C_2-Li_2S_8（e）d-Ti_3C_2-Li_2S_8-2（f）d-Ti_3C_2-S_8

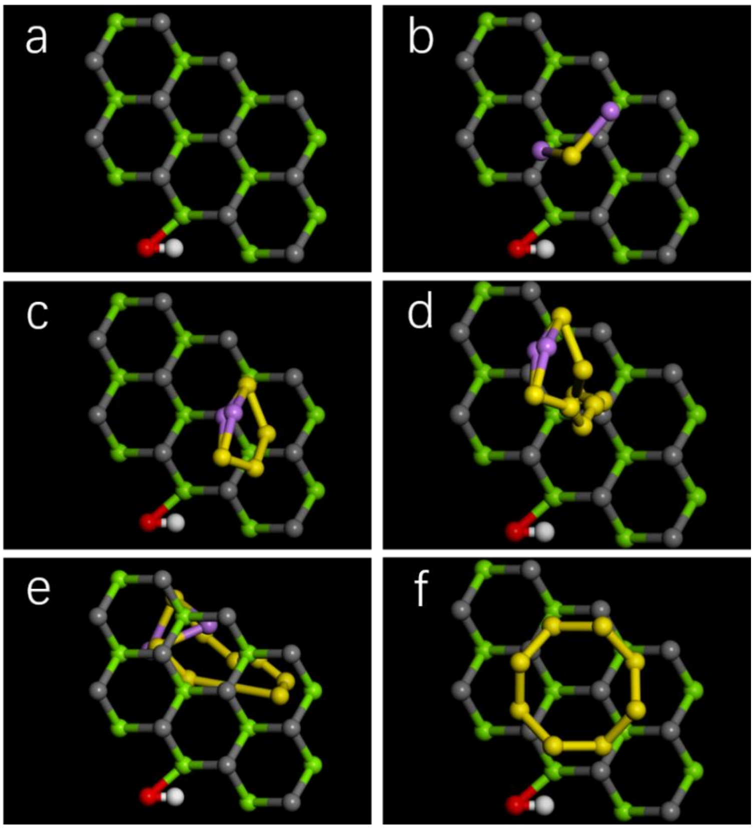

Figure S24 Schematic representation of the molecular structure of externally attached hydroxyhexacyclic d-Ti_3C_2 and its adsorbed lithium polysulfide: （a）d-Ti_3C_2（b）d-Ti_3C_2-Li_2S（c）d-Ti_3C_2-Li_2S_4（d）d-Ti_3C_2-Li_2S_8（e）d-Ti_3C_2-Li_2S_8-2（f）d-Ti_3C_2-S_8

Table S1 Summary of the binding energies of Gr to polysulphides

Category	Energy/eV	Category	Energy/eV
LiS	-3.144	Li_2S	-3.704
Li_2S_4	-2.922	Li_2S_8	-3.590
Li_2S_8-2	-1.980	S_8	-2.714

Table S2 Summary of the binding energies of Gr and polysulphides with externally attached carboxyl groups at the pyridine position

Category	Energy/eV	Category	Energy/eV
LiS	-2.188	Li_2S	-2.529

Li_2S_4	-1.825	Li_2S_8	-1.870
Li_2S_8-2	-0.795	S_8	-0.789

Table S3 Summary of the binding energies of Gr and polysulfides with hydroxyl and carboxyl groups externally attached to the pyridine position

name	Energy/eV	name	Energy/eV
LiS	-2.281	Li_2S	-1.516
Li_2S_4	-0.930	Li_2S_8	-1.222
Li_2S_8 -2	-1.944	S_8	-0.714

Table S4 Summary of the binding energies of Gr and polysulphides with externally attached carboxyl groups at the pyrrole site

name	Energy/eV	name	Energy/eV
LiS	-2.162	Li_2S	-5.889
Li_2S_4	-1.866	Li_2S_8	-1.848
Li_2S_8-2	-1.629	S_8	12.804

Table S5 Summary of the binding energies of graphite sites externally connected to bis-hydroxy Gr and polysulphides

name	Energy/eV	name	Energy/eV
LiS	-5.301	Li_2S	-2.770
Li_2S_4	-3.997	Li_2S_8	-2.592
Li_2S_8 -2	-1.380	S_8	-4.064

Table S6 Summary of the binding energies of Gr and polysulphides with hydroxyl and carboxyl groups externally attached to graphite sites

name	Energy/eV	name	Energy/eV
LiS	-5.973	Li_2S	-6.540
Li_2S_4	-2.198	Li_2S_8	-2.438
Li_2S_8-2	0.438	S_8	-1.226

Table S7 Summary of the binding energies of LiS to graphene and graphene oxide

name	Energy/eV	name	Energy/eV
Gr	-3.144	Gr (Carboxylic acid attached to the pyridine position)	-2.188
Gr (Pyridine position attached to hydroxyl and carboxyl groups)	-2.281	Gr (The pyrrole position is connected to the carboxyl group)	-2.162
Gr (Graphite Bit Attached to Double Hydroxyl)	-5.301	Gr (Graphite sites connected to hydroxyl and carboxyl)	-5.973

Table S8 Summary of the binding energies of Li_2S to graphene and graphene oxide

name	Energy/eV	name	Energy/eV
Gr	-3.704	Gr (Carboxylic acid attached to the pyridine position)	-2.529
Gr (Pyridine position attached to hydroxyl and carboxyl groups)	-1.516	Gr (The pyrrole position is connected to the carboxyl group)	-5.889
Gr (Graphite Bit Attached to Double Hydroxyl)	-2.770	Gr (Graphite sites connected to hydroxyl and carboxyl)	-6.540

Table S9 Summary of the binding energies of Li_2S_4 to graphene and graphene oxide

name	Energy/eV	name	Energy/eV

Gr	-2.992	Carboxylic acid Gr externally attached to the pyridine position	-1.825
Pyridine position externally attached to hydroxyl and carboxyl groups Gr	-0.930	The pyrrole position is externally connected to the carboxyl group Gr	-1.866
Graphite Bit Externally Attached to Double Hydroxyl Gr	-3.997	Graphite sites externally connected to hydroxyl and carboxyl Gr	-2.198

Table S10 Summary of the binding energies of Li_2S_8 to graphene and graphene oxide

name	Energy/eV	name	Energy/eV
Gr	-3.590	Carboxylic acid Gr externally attached to the pyridine position	-1.870
Pyridine position externally attached to hydroxyl and carboxyl groups Gr	-1.222	The pyrrole position is externally connected to the carboxyl group Gr	-1.848
Graphite Bit Externally Attached to Double Hydroxyl Gr	-2.592	Graphite sites externally connected to hydroxyl and carboxyl Gr	-2.438

Table S11 Summary of the binding energies of Li_2S_8-2 to graphene and graphene oxide

name	Energy/eV	name	Energy/eV
Gr	-1.980	Carboxylic acid Gr externally attached to the pyridine position	-0.795

Pyridine position externally attached to hydroxyl and carboxyl groups Gr	-1.944	The pyrrole position is externally connected to the carboxyl group Gr	-1.629
Graphite Bit Externally Attached to Double Hydroxyl Gr	-1.380	Graphite sites externally connected to hydroxyl and carboxyl Gr	0.438

Table S12 Summary of the binding energies of S_8 to graphene and graphene oxide

name	Energy/eV	name	Energy/eV
Gr	-2.174	Carboxylic acid Gr externally attached to the pyridine position	-0.789
Pyridine position externally attached to hydroxyl and carboxyl groups Gr	-0.714	The pyrrole position is externally connected to the carboxyl group Gr	12.804
Graphite Bit Externally Attached to Double Hydroxyl Gr	-4.064	Graphite sites externally connected to hydroxyl and carboxyl Gr	-1.226

Table S13 Summary of the binding energies of DOL to polysulphides

name	Energy/eV	name	Energy/eV
DOL-LiS	-1.181	DOL- Li_2S	-1.178
DOL-Li_2S_4	-0.225	DOL-Li_2S_8	-1.871
DOL-Li_2S_8-2	-1.234	DOL-S_8	-0.431

Table S14 Summary of the binding energies of DME to polysulphides

name	Energy/eV	name	Energy/eV
DME-LiS	-1.157	DME- Li_2S	-1.054

DME-Li_2S_4	-1.145	DME-Li_2S_8	-0.427
DME-Li_2S_8-2	-0.588	DME-S_8	-0.341

Table S15 Summary of the binding energies of ionic liquids N1113+-PF6- to polysulphides

name	Energy/eV	name	Energy/eV
N1113+-PF6--LiS	-3.665	N1113+-PF6--Li_2S	-3.707
N1113+-PF6-- Li_2S_4	-6.095	N1113+-PF6--Li_2S_8	-5.525
N1113+-PF6--Li_2S_8 -2	-1.790	N1113+-PF6--S_8	-0.167

Table S16 Summary of the binding energies of ionic liquid N3333+-PF6- to polysulphides

name	Energy/eV	name	Energy/eV
N3333+-PF6--LiS	0.872	N3333+-PF6--Li_2S	-2.382
N3333+-PF6- Li_2S_4	-2.954	N3333+-PF6--Li_2S_8	-2.798
N3333+-PF6--Li_2S_8 -2	-1.822	N3333+-PF6--S_8	-5.861

Table S17 Summary of the binding energies of ionic liquids P13+-PF6- to polysulphides

name	Energy/eV	name	Energy/eV
P13+-PF6--LiS	-5.393	P13+-PF6-- Li_2S	-3.993
P13+-PF6--Li_2S_4	-1.733	P13+-PF6--Li_2S_8	0.614
P13+-PF6--Li_2S_8-2	-6.090	P13+-PF6--S_8	-3.276

Table S18 Summary of the binding energies of ionic liquids PMIM+-BF4- to

polysulphides

name	Energy/eV	name	Energy/eV
PMIM+-BF4--LiS	-4.245	PMIM+-BF4-- Li_2S	-6.508
PMIM+-BF4--Li_2S_4	-1.924	PMIM+-BF4--Li_2S_8	-1.478
PMIM+-BF4--Li_2S_8-2	-3.914	PMIM+-BF4--S_8	-1.240

Table S19 Summary of the binding energies of ionic liquids PP13+-TFSI- to polysulphides

name	Energy/eV	name	Energy/eV
PP13+-TFSI--LiS	-6.626	P13+- TFSI-- Li_2S	-7.376
PP13+- TFSI--Li_2S_4	-5.896	PP13+- TFSI-- Li_2S_8	-6.829
PP13+- TFSI--Li_2S_8 -2	0.288	PP13+- TFSI--S_8	-0.126

Table S20 Summary of the binding energies of ionic liquids Py3+-PF6- to polysulfides

name	Energy/eV	name	Energy/eV
Py3+-PF6--LiS	-2.198	Py3+-PF6-- Li_2S	-3.375
Py3+-PF6- Li_2S_4	-1.508	Py3+-PF6--Li S_{28}	-4.746
Py3+-PF6--Li S_{28} -2	-4.115	Py3+-PF6--S_8	-1.987

Table S21 Summary of the binding energies of LiS to ionic liquids and common solvents

name	Energy/eV	name	Energy/eV
DOL	-1.181	DME	-1.157
N1113+-PF6-	-3.665	N3333+-PF6-	0.872
P13+-PF6-	-5.393	PMIM+-BF4-	-4.245
PP13+-TFSI-	-6.626	Py3+-PF6-	-2.198

Table S22 Summary of the binding energies of Li_2S to ionic liquids and common solvents

name	Energy/eV	name	Energy/eV
DOL	-1.178	DME	-1.054
N1113+-PF6-	-3.707	N3333+-PF6-	-2.382
P13+-PF6-	-3.993	PMIM+-BF4-	-6.508
PP13+-TFSI-	-7.376	Py3+-PF6-	-3.375

Table S23 Summary of the binding energies of Li_2S_4 to ionic liquids and common solvents

name	Energy/eV	name	Energy/eV
DOL	-0.225	DME	-1.145
N1113+-PF6-	-6.095	N3333+-PF6-	-2.954
P13+-PF6-	-1.733	PMIM+-BF4-	-1.924
PP13+-TFSI-	-5.896	Py3+-PF6-	-1.508

Table S24 Summary of the binding energies of Li_2S_8 to ionic liquids and common solvents

name	Energy/eV	name	Energy/eV
DOL	-1.871	DME	-0.427
N1113+-PF6-	-5.525	N3333+-PF6-	-2.798
P13+-PF6-	0.614	PMIM+-BF4-	-1.478
PP13+-TFSI-	-6.829	Py3+-PF6-	-4.746

Table S25 Summary of the binding energies of Li_2S_8-2 with ionic liquids and common solvents

name	Energy/eV	name	Energy/eV
DOL	-1.234	DME	-0.588
N1113+-PF6-	-1.790	N3333+-PF6-	-1.822
P13+-PF6-	-6.090	PMIM+-BF4-	-3.914

PP13+-TFSI-	0.288	Py3+-PF6-	-4.115

Table S26 Summary of the binding energies of S_8 to ionic liquids and common solvents

name	Energy/eV	name	Energy/eV
DOL	-0.431	DME	-0.314
N1113+-PF6-	-0.167	N3333+-PF6-	-5.861
P13+-PF6-	-3.276	PMIM+-BF4-	-1.240
PP13+-TFSI-	-0.126	Py3+-PF6-	-1.987

Table S27 Summary of the binding energies of decacyclic d-Ti_3C_2 to polysulphides

name	Energy/eV	name	Energy/eV
d-Ti_3C_2-Li S_2	-4.793	d-Ti_3C_2-Li_2S_4	-7.466
d-Ti_3C_2- Li_2S_8	-12.092	d-Ti_3C_2-Li_2S_8-2	-14.659
d-Ti_3C_2-S_8	-12.240		

Table S28 Summary of the binding energies of hexacyclic d-Ti_3C_2 to polysulfides

name	Energy/eV	name	Energy/eV
d-Ti_3C_2- Li_2S	-6.532	d-Ti_3C_2- Li_2S_4	-9.691
d-Ti_3C_2- Li_2S_8	-10.136	d-Ti_3C_2- Li_2S_8-2	-17.733
d-Ti_3C_2-S_8	-17.383		

Table S29 Summary of the binding energies of mono-C-deficient hexacyclic d-Ti_3C_2 to polysulfides

name	Energy/eV	name	Energy/eV
d-Ti_3C_2- Li_2S	-4.546	d-Ti_3C_2- Li_2S_4	-5.248
d-Ti_3C_2-V	-9.560	d-Ti_3C_2- Li_2S_8-2	-13.414
d-Ti_3C_2-S_8	-15.828		

Table S30 Summary of the binding energies of mono-Ti defective hexacyclic d-Ti_3C_2 to polysulfides

name	Energy/eV	name	Energy/eV
d-Ti_3C_2 - Li_2S	-3.220	d-Ti_3C_2- Li_2S_4	-7.685
d-Ti_3C_2- Li_2S_8	-14.630	d-Ti_3C_2- Li_2S_8-2	-5.033
d-Ti_3C_2-S_8	-10.379		

Table S31 Summary of the binding energies of externally connected F-atom hexacyclic d-$Ti_3C_2$to polysulfides

name	Energy/eV	name	Energy/eV
d-Ti_3C_2- Li_2S	-1.445	d-Ti_3C_2- Li_2S_4	-8.563
d-Ti_3C_2- Li_2S_8	-8.240	d-Ti_3C_2- Li_2S_8-2	-9.931
d-Ti_3C_2-S_8	-8.261		

Table S32 Summary of the binding energies of externally attached hydroxyhexacyclic d-Ti_3C_2 to polysulfides

name	Energy/eV	name	Energy/eV
d-Ti_3C_2- Li_2S	-1.194	d-Ti_3C_2- Li_2S_4	-5.727
d-Ti_3C_2- Li_2S_8	-7.299	d-Ti_3C_2- Li_2S_8-2	-7.572
d-Ti_3C_2-S_8	-10.163		

Table S33 The binding energy of Li_2S with d-Ti_3C_2 MXene

name	energy/eV	name	energy/eV
Decacyclic d-Ti_3C_2	-4.793	Unary Ti hexacyclic d-Ti_3C_2	-3.220
Hexacyclic d-Ti_3C_2	-6.532	External F atom hexacyclic d-Ti_3C_2	-1.445
Unary C hexacyclic d-Ti_3C_2	-4.546	External hydroxyl hexacyclic	-1.194

Table S34 The binding energy of Li_2S_8 with d-Ti_3C_2 MXene

name	energy/eV	name	energy/eV
Decacyclic d-Ti_3C_2	-12.092	Unary Ti hexacyclic d-Ti_3C_2	-14.630
Hexacyclic d-Ti_3C_2	-10.136	External F atom hexacyclic d-Ti_3C_2	-8.240
Unary C hexacyclic d-Ti_3C_2	-9.560	External hydroxyl hexacyclic	-7.299

Table S35 The binding energy of S_8 with d-Ti_3C_2 MXene

name	energy/eV	name	energy/eV
Decacyclic d-Ti_3C_2	-12.240	Unary Ti hexacyclic d-Ti_3C_2	-10.379
Hexacyclic d-Ti_3C_2	-17.383	External F atom hexacyclic d-Ti_3C_2	-8.261
Unary C hexacyclic d-Ti_3C_2	-15.828	External hydroxyl hexacyclic	-10.163

Table S36 The binding energy of Li_2S_4 with d-Ti_3C_2 MXene

name	energy/eV	name	energy/eV
Decacyclic d-Ti_3C_2	-7.466	Unary Ti hexacyclic d-Ti_3C_2	-7.685
Hexacyclic d-Ti_3C_2	-9.691	External F atom hexacyclic d-Ti_3C_2	-8.563
Unary C hexacyclic d-Ti_3C_2	-5.248	External hydroxyl hexacyclic	-5.727

Table S37 The binding energy of Li_2S_8-2 with d-Ti_3C_2 MXene

name	energy/eV	name	energy/eV
Decacyclic d-Ti_3C_2	-14.659	Unary Ti hexacyclic d-Ti_3C_2	-5.033
Hexacyclic d-Ti_3C_2	-17.733	External F atom hexacyclic d-Ti_3C_2	-9.931
Unary C hexacyclic d-Ti_3C_2	-13.414	External hydroxyl hexacyclic	-7.572
